# A Parsimonious Machine Learning Model for Predicting Invasive Coronary Angiography Referral After Coronary Computed Tomography Angiography: Development and Multicentre External Validation

**DOI:** 10.3390/jcm15197641

**Published:** 2026-10-02

**Authors:** Alec Chunta, Caitlyn Gilbert, Dina Labib, Augustine Amakiri, Yuanchao Feng, Atefeh Alavi Fili, Derek Chew, Benjamin JW Chow, Michelle Graham, Julio Garcia, Mariana Bento, James White, Robert JH Miller

**Affiliations:** 1 Departments of Radiology and Cardiac Sciences, Libin Cardiovascular Institute, Cumming School of Medicine, University of Calgary, Calgary, AB T2N 4Z6, Canada; alec.chunta1@ucalgary.ca (A.C.);; 2 Department of Biomedical Engineering, Schulich School of Engineering, University of Calgary, Calgary, AB T2N 4Z6, Canada; 3 Department of Medicine (Division of Cardiology and Nuclear Medicine), University of Ottawa Heart Institute, Ottawa, ON K1Y 4W7, Canada; 4 Department of Medicine and Mazankowski Heart Centre, University of Alberta, Edmonton, AB T6G 0M9, Canada

**Keywords:** coronary artery disease, coronary computed tomography angiography, invasive coronary angiography, machine learning, extreme gradient boosting, feature selection, parsimonious model, clinical prediction model, external validation, SHAP

## Abstract

**Background/Objectives:** Coronary computed tomography angiography (CCTA) is recommended as a first-line test for suspected coronary artery disease. Many patients undergo invasive coronary angiography (ICA) after CCTA, and no prediction model exists to estimate the probability of this downstream testing. We aimed to develop and validate a parsimonious extreme gradient boosting (XGB) model for predicting ICA referral after CCTA. **Methods:** Adults undergoing a CCTA were identified at three Canadian centres. An XGB model was trained on 156 variables in the internal cohort (Calgary, *n* = 8823) and tested in an external cohort (Edmonton, *n* = 10,596). The full model and a reduced 15-variable model were compared using discrimination and calibration. **Results:** ICA referral occurred in 1161 (13.2%) internal and 1269 (12.0%) external patients. Internally, the reduced model matched the full model (AUC 0.698 vs. 0.701; *p* = 0.16) with equivalent calibration (Brier 0.1077 vs. 0.1076; slope 1.21 vs. 1.18). Externally, the full model retained a small but significant advantage (AUC 0.701 vs. 0.687; *p* < 0.001). Age, anginal nitrate use, and lowest HDL cholesterol were the strongest predictors. **Conclusions:** A 15-variable XGB model preserved most of the predictive performance of a 156-variable model for ICA referral after CCTA. Clinical use would require evidence that it improves patient selection for ICA.

## 1. Introduction

Coronary artery disease (CAD) remains a leading cause of mortality and morbidity worldwide [1]. Current guidelines recommend a diagnostic pathway for suspected chronic coronary syndrome in which the choice of initial test is guided by the estimated clinical likelihood of obstructive CAD [2,3]. Coronary computed tomography angiography (CCTA) is recommended in patients with low-to-moderate clinical likelihood, in whom it can reliably rule out obstructive CAD and identify non-obstructive atherosclerosis that warrants preventive therapy [3,4]. Direct invasive coronary angiography (ICA) is reserved for patients with a very high clinical likelihood, high-risk clinical features, or symptoms refractory to medical therapy, in whom invasive assessment and possible revascularisation are anticipated [3]. In a randomised comparison of these strategies in patients with intermediate pre-test probability, initial CCTA resulted in a similar rate of major adverse cardiovascular events and fewer major procedure-related complications than initial ICA [5].

CCTA and ICA are therefore complementary rather than interchangeable. Proceeding to ICA after CCTA is appropriate when CCTA identifies obstructive or high-risk disease, or when it is non-diagnostic. When a patient who would have met criteria for direct invasive assessment first undergoes CCTA, however, the result is two contrast-enhanced, radiation-exposing procedures where one might have sufficed [6]. Approximately 23% of patients undergo ICA after CCTA [7,8]. Identifying, at the time of CCTA referral, the patients most likely to proceed to ICA could help align the choice of initial test with guideline recommendations. Existing pre-test tools, including the ESC risk-factor-weighted clinical likelihood model and the CAD Consortium score, estimate the probability of obstructive CAD rather than the probability of downstream invasive testing, and no model currently estimates the latter [3,9].

Machine learning (ML) has emerged as a powerful tool for clinical prediction and can surpass traditional methods by effectively integrating diverse patient variables. ML can capture high-order interactions and non-linear relationships between variables, which may not be possible with conventional statistical methods [10]. Approaches similar to this have been used to predict the risk of major cardiovascular events, identify patients with CAD, and detect early disease [11,12].

Prediction potential alone, however, does not make a model practical in a clinical setting. A model that depends on more than one hundred variables is difficult to implement into a referral workflow. Therefore, the practical value of a prediction model not only depends on how well it predicts but also how little information is required. Reducing the number of input variables addresses barriers related to model usability and deployment. Reducing the feature set lowers the data burden and improves transparency since the model is built on a small set of features physicians know and trust. These improvements are only worthwhile if the reduced model can preserve the performance of the full model. This research explores whether a full XGB model can be reduced to a clinically accessible feature set without meaningful performance loss. Utilizing gain-based feature importance, correlation filtering and sequential feature addition, we create a parsimonious model and compare its discrimination, calibration, and external generalizability with the full model.

## 2. Materials and Methods

### 2.1. Study Design and Data Sources

This retrospective, multicentre cohort study included patients undergoing a clinically indicated CCTA at three Canadian sites. Patient data from the University of Calgary were used for model development, internal training and validation. Data from the University of Alberta (Edmonton) were used exclusively for external testing. Additionally, secondary external testing was undertaken using a cohort of patients from the University of Ottawa Heart Institute (see Supplementary Material). This cohort allowed us to evaluate differences in data accessibility and our model’s ability to generalize to different health systems across Canada. The Calgary dataset was chosen as the derivation cohort as it was the first of the three datasets available for analysis, with the Edmonton and Ottawa cohorts reserved as independent external test sets. This structure supports assessment of model transportability to health systems not represented during training. This study received approval through the institutional review boards at each site.

### 2.2. Study Population

The patient population for this study included adult patients who were referred for an index CCTA anytime between 1 January 2016, and 31 December 2024, at participating institutions. No patients were excluded during the study time frame. If patients had more than one CCTA during the study time frame, only the first patient record was included.

Before data cleaning and pre-processing, there were a total of 30,080 patients across the three sites: 9082 from the University of Calgary, 10,905 from the University of Alberta, and 10,093 from the University of Ottawa. After duplicate patient encounters, blank outcomes, and blank sex rows were removed from the dataset, there were 29,278 patients overall: 8823 from Calgary, 10,596 from Edmonton, and 9859 from Ottawa.

### 2.3. Outcome

The primary outcome of interest in this study is referral for ICA within 90 days of CCTA testing. This outcome reflects a clinical care pathway decision and was not intended as a measure of obstructive CAD, hemodynamically significant disease, revascularisation, or the appropriateness of ICA. The chosen threshold of 90 days aligns with typical clinical workflow, as ICA referrals based on actionable findings typically occur within a 90-day time frame.

The 90-day window was selected to encompass the interval over which ICA is typically performed in response to CCTA findings, while limiting the addition of procedures prompted by subsequent clinical events. Cardiac catheterization procedures in Alberta are recorded in the APPROACH registry, which captures procedures performed at provincial sites irrespective of the centre at which the index CCTA was undertaken, and ICA status was ascertained for every patient in the internal and external cohorts. Patients with missing outcome data were removed during data cleaning, as described in Section 2.2.

### 2.4. Predictor Variables

In this study, 156 variables were used to train the models, and 49 were used in the secondary testing (see Table S1 for a comprehensive list of variables used in this work). Predictor variable categories included medical history, laboratory, medication and outcome data referenced to the index CCTA. These variables were obtained from institutional and provincial databases using established ICD-10 codes [13]. Cardiovascular risk factors, cardiovascular and non-cardiovascular comorbidities, outcomes, and procedural history were obtained from provincial, administrative databases, including the discharge abstract database and the National Ambulatory Care Reporting System. Provincial laboratory databases such as Alberta Laboratory Services were used to collect common laboratory markers up to 3 years prior to the date of the CCTA, preferentially including the value closest to the time of CCTA [14,15]. This lookback period reflects the structure of provincial laboratory data, in which testing occurs at clinically indicated intervals rather than at fixed times before imaging; restricting to a narrower window would have excluded patients without recent testing and introduced missingness related to healthcare contact rather than to the outcome. In Alberta, medication data was obtained from the Pharmaceutical Information Network (PIN), which captures >95% of prescriptions [16], based on prescription fills in the 180 days prior to CCTA. Data on cardiovascular procedures prior to the index CCTA were obtained from the APPROACH registry. Variables that would not be available at the time of CCTA referral or that reflected downstream diagnostic procedures (e.g., angiographic findings) were excluded from analysis to prevent post-index information data leakage. A summary of dataset sources is outlined in Table S2. All predictors were restricted to information available at the time of CCTA referral. CCTA findings, including stenosis severity, CAD-RADS category, plaque burden and high-risk anatomy, were not included because they were not available in structured form across all three sites. The model is therefore intended for use at the point of CCTA referral to estimate the probability that a patient will proceed to ICA, rather than as decision support after CCTA has been interpreted.

### 2.5. Data Pre-Processing

Data pre-processing was applied consistently across all cohorts prior to model development to ensure reproducibility and prevent information leakage.

Patient data from the internal cohort were abstracted and pseudonymized under the PULSE framework, which applies unique pseudonymous identifiers while preserving consistency across data sources [17]. External datasets underwent equivalent pseudonymization prior to transfer.

Variables that represented related diagnostic categories (e.g., diabetes mellitus type 1 and 2) were consolidated into composite (combined) variables to improve clinical interpretability. Combined variables were defined using strict rule-based criteria that incorporated ICD-10 codes, medication exposure and laboratory values prior to the index CCTA [18]. After all pre-processing steps were complete the internal dataset consisted of 156 variable predictors.

The overall proportion of missing values within the internal cohort was 5.9% and the external cohort was 10.2%; this represents the total missing data relative to the size of the entire dataset. The missingness percentage of each variable used in the dataset is available in Table S3. See Table S4 for missingness within secondary external testing.

### 2.6. Extreme Gradient Boosting

The XGB model was implemented using the *xgboost* package (version 1.7.11.1). XGB uses gradient-boosted decision trees in which trees are trained sequentially, and each subsequent tree is fit to minimize the residual errors of the prior tree. XGBoost handles missing values natively by learning optimal split directions for missing data during tree construction, eliminating the need for prior imputation. Hyperparameters for the model were optimized using a grid search approach and internal cross-validation.

### 2.7. Model Training and Validation

**Figure 1:**
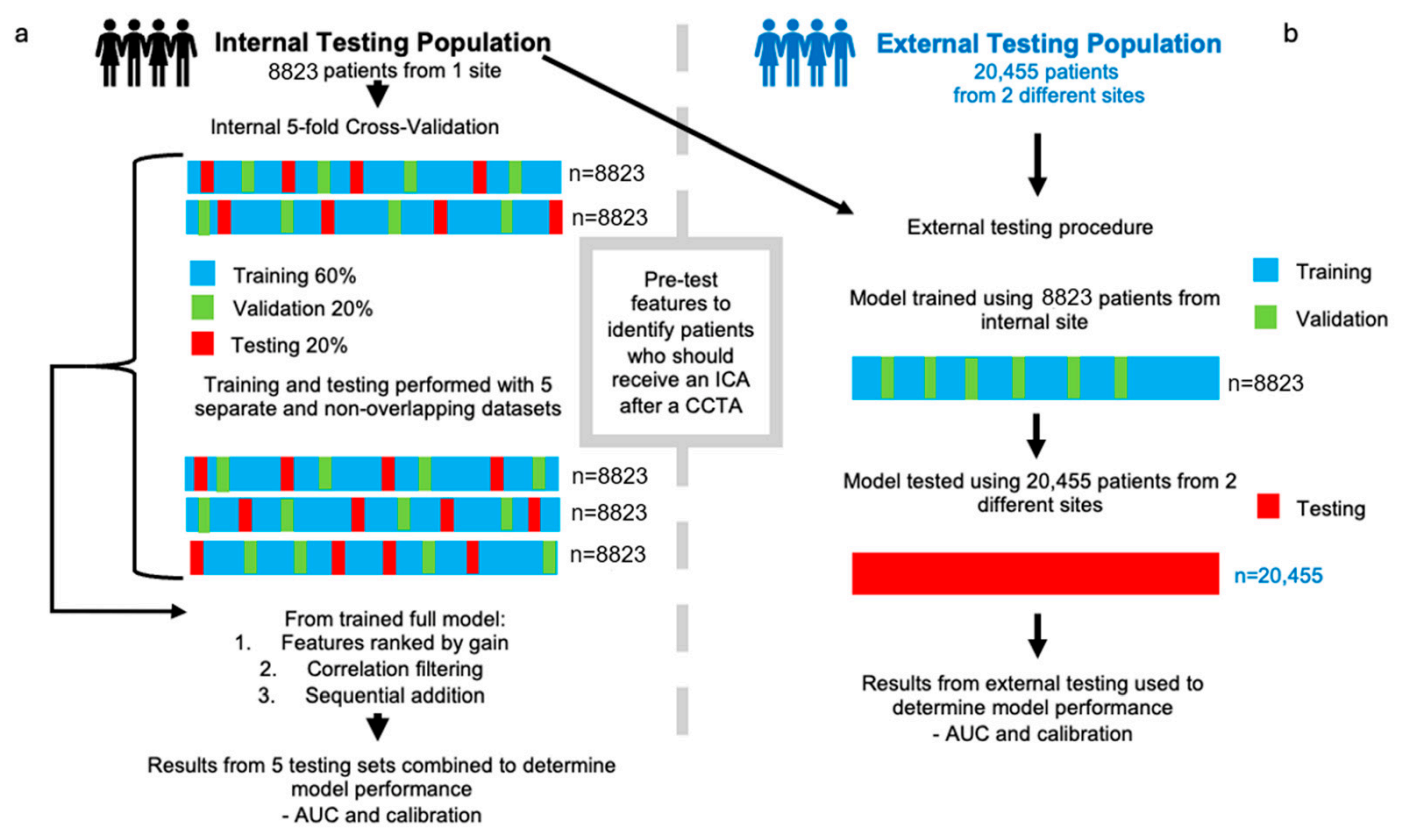
Internal training and external testing procedures for the full and parsimonious models. Schematic of the model development and validation pipeline. (**a**) Internal validation in the Calgary cohort used stratified five-fold cross-validation; in each iteration, three folds were used for training, one for tuning, and one for testing, with each fold serving as the test set once. Gain-based feature importance was extracted, correlated features (Pearson r > 0.85) were removed, and retained features were added sequentially to build the reduced model; performance was pooled across the five held-out test folds. (**b**) For external validation, the full and reduced models were trained on Calgary and tested in two independent cohorts (Edmonton and Ottawa).

Model development and internal validation were performed using the internal dataset. Model development utilized a single stratified 5-fold partition of the internal dataset. At each of the five iterations, three folds (60%) were used for model training, one fold (20%) for tuning, and one fold (20%) for model testing, with the tuning and testing folds rotating so that every fold served as the testing set once (see Figure 1). Within each iteration, hyperparameters were selected using performance on the tuning fold alone; the testing fold contributed to neither model fitting nor hyperparameter selection and was used solely to generate held-out predictions. This design was chosen so that all internal data contributed to model fitting and evaluation before the final model was refit on the full internal cohort.

No resampling or class weighting was applied to the model at any point during training. Synthetic oversampling techniques such as SMOTE can distort the predicted probability distribution and degrade calibration, which is important to this application as the intended use is risk communication rather than hard disease classification. Interpolated synthetic patients may produce profiles that are not representative of the minority class when created with a complex medical dataset [19]. Furthermore, resampling and class weighting would raise the apparent referral rate, overestimating risk predictions. An over-stated probability would artificially inflate sensitivity values, and for the purpose of this study, base rate model performance was prioritized over inflated statistical metrics [20], particularly because an over-inflated referral probability in this setting could contribute to an invasive procedure that the patient did not require.

For external testing, the full internal Calgary dataset was used to train the final model using the previously identified hyperparameters. The resulting model was then applied without re-tuning to the Edmonton external cohort, and separately to the Ottawa cohort. External cohorts were held out during all stages of model training, feature development and hyperparameter tuning.

### 2.8. Feature Importance Ranking

Following internal model training on the full variable set, feature importance was extracted from the trained XGB model using the built-in gain metric (information gain). This metric quantifies the average improvement in model loss attributable to each feature across all splits [21]. Gain-based importance was used as the primary ranking tool for feature selection in the parsimonious model, as it reflects the direct predictive contribution of each variable to the outcome, rather than its frequency of use in splitting decisions. Features were ranked in descending order of mean gain across the five-fold cross-validation to ensure that importance estimates were stable and not driven by a single data partition.

### 2.9. Correlation Filtering

After the initial feature importance, and prior to parsimonious model construction, a pairwise correlation analysis was conducted across all variables using Pearson’s correlation coefficients. A correlation heatmap was generated to visualize inter-feature relationships (see Figures S1 and S2). Variable pairs exceeding a correlation threshold of r > 0.85 were identified and the lower-ranked variable within each correlated pair, as determined by the initial XGB gain-based feature importance, was removed [22]. This step was applied to reduce multicollinearity and prevent redundant features from inflating importance rankings or obscuring the contribution of clinically distinct predictors in the parsimonious model [23].

### 2.10. Sequential Feature Addition

A sequential feature addition procedure was conducted to identify the minimum sufficient feature set for ICA prediction. Beginning with the single highest-ranked feature by gain, one feature was added at each step in descending order of importance, and a new XGB model was trained on the updated feature set. This process was repeated until the top 15 variables were incorporated, resulting in a series of models, each trained on one additional variable relative to the prior step. The parsimonious model was pre-specified as the 15 highest gain-ranked features retained after correlation filtering, and this feature set was unchanged and applied to both the internal and external cohorts to ensure comparability. The sequential curve was used to verify the capability of the model rather than to determine its size. AUC gains below 0.005 per added variable indicated that the performance had plateaued within the 15-variable cap. Hyperparameters for the full model were used for the parsimonious model after feature reduction.

### 2.11. SHAP Analysis

By using SHapley Additive exPlanations (SHAP), we were able to provide a consistent model-agnostic characterization of each feature’s contribution to predictions across all patients in the dataset. SHAP values decompose each model prediction into additive contributions and satisfy desirable properties including accuracy, missingness, and consistency based on cooperative game theory [24]. SHAP values in this study were used exclusively for model interpretation and clinical contextualization of the final parsimonious model. SHAP’s use as an interpretation tool was decided due to its ability to map patient-level attributions that gain-based importance cannot supply. It was applied to the external cohorts to assess whether the reduced model’s reasoning transferred to independent populations rather than reflecting patterns specific to the internal site. Results were visualized using a SHAP beeswarm plot which communicates feature importance ranking, the distribution of SHAP values across patients, and the direction of each feature’s effect on the predicted probability of ICA referral.

### 2.12. Statistical Analysis

Categorical variables were summarized as numbers (proportions) and compared with a Chi-square or Fisher exact test as appropriate. Continuous variables were summarized as mean (standard deviation) and compared with Student’s *t*-test if normally distributed, and with a Wilcoxon rank-sum test if not. Receiver operating characteristic (ROC) curves were constructed for each model. Discriminative performance was compared using area under the ROC curve (AUC), with comparisons between models performed using the non-parametric method proposed by DeLong et al. [25]. Optimal classification thresholds were derived using Youden’s index with the internal cohorts and then applied without re-tuning to the external cohorts. At the Youden-derived thresholds, sensitivity, specificity, positive predictive value (PPV), negative predictive value (NPV), and F1 score were calculated. McNemar’s test was used to compare paired sensitivity and specificity scores. Paired NPV, PPV, and F1 scores were compared using paired bootstrap resampling with 10,000 replications [26]. To assess model calibration, Brier scores and calibration graphs were used. Brier scores were compared for statistical significance using a paired two-sample *t*-test where each observation’s squared difference between predicted probability and observed outcome was treated as the unit of comparison. Calibration was additionally characterized by the calibration intercept and calibration slope, estimated from a logistic recalibration model which relates observed outcomes to the predicted linear predictor using base R with 95% confidence intervals obtained by bootstrap resampling.

All statistical tests used in our study were two-sided, and a *p*-value of <0.05 was considered significant. Statistical analysis was completed using R version 4.5.3.

### 2.13. Decision Curve Analysis

To assess clinical utility, decision curve analysis was performed for the full and reduced models in the internal and external cohorts [27]. The clinical decision was defined as whether a patient being considered for CCTA should instead proceed directly to ICA. Net benefit was calculated across threshold probabilities from 0 to 0.95 and compared with two default strategies: referring all patients for direct ICA and referring none. The threshold probability represents the point at which a clinician is indifferent between the two actions, and therefore encodes the relative harm assigned to false-positive and false-negative classifications: a threshold of *p*_t_ implies that one false negative is considered equivalent to (1 − p_t_)/p_t_ false positives [28]. Internal decision curves used the pooled out-of-fold predictions from the five cross-validation test folds. Classification consequences per 1000 patients were reported at thresholds of 5%, 10%, 15%, 20% and 30%, and at the Youden-derived threshold described in Section 2.12. Analyses used the *dcurves* package (version 0.5.1) in R.

## 3. Results

### 3.1. Study Population

Characteristics of the internal and external validation cohorts are presented in Table 1 for Calgary and Edmonton. The internal cohort included 8823 patients from Calgary (median age 59 years, IQR 49–66; 57.6% male), with hypertension in 36.1%, diabetes in 23.0%, and dyslipidemia in 75.6%. Of those patients, 1161 (13.2%) underwent ICA within 90 days. Compared with Calgary, the Edmonton external cohort (*n* = 10,596; median age 57, IQR 49–65; 54.1% male) was slightly younger and had lower recorded rates of hypertension (35.8%) and dyslipidemia (60.0%), but a greater number of patients with diabetes (24.0%). In the external cohort, 1269 (12.0%) underwent a subsequent ICA. Table S5 compares patient populations from Calgary and Ottawa. The Ottawa external cohort (*n* = 9859; median age 59, IQR 51–67; 55.7% male) had similar age and sex distribution, but lower diabetes (14.8%) and dyslipidemia (48.0%), higher hypertension (48.9%), and a smaller number of patients, 294 (3.0%), undergoing a subsequent ICA.

### 3.2. Internal Results

**Figure 2:**
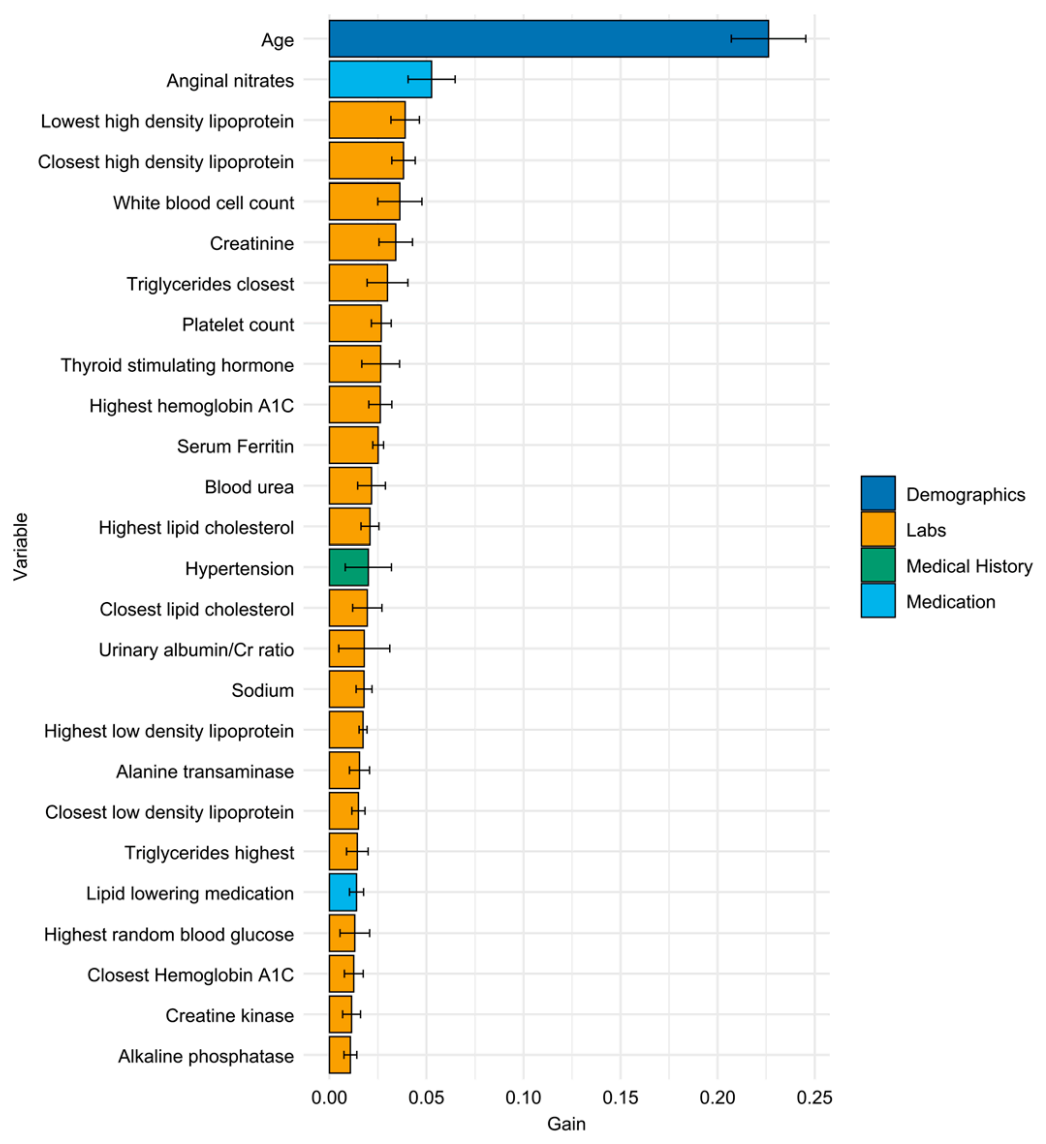
Gain-based feature importance in the internal cohort (Calgary). Mean gain-based importance for each variable in the full XGBoost model, ranked in descending order across the five cross-validation folds. Gain quantifies the average improvement in model loss attributable to a feature across all splits in which it is used. The three highest-ranked predictors of subsequent ICA were age, anginal nitrate usage, and lowest HDL cholesterol. Abbreviations: CAD, coronary artery disease; HDL, high-density lipoprotein; ICA, invasive coronary angiography; XGB, extreme gradient boosting.

**Figure 3:**
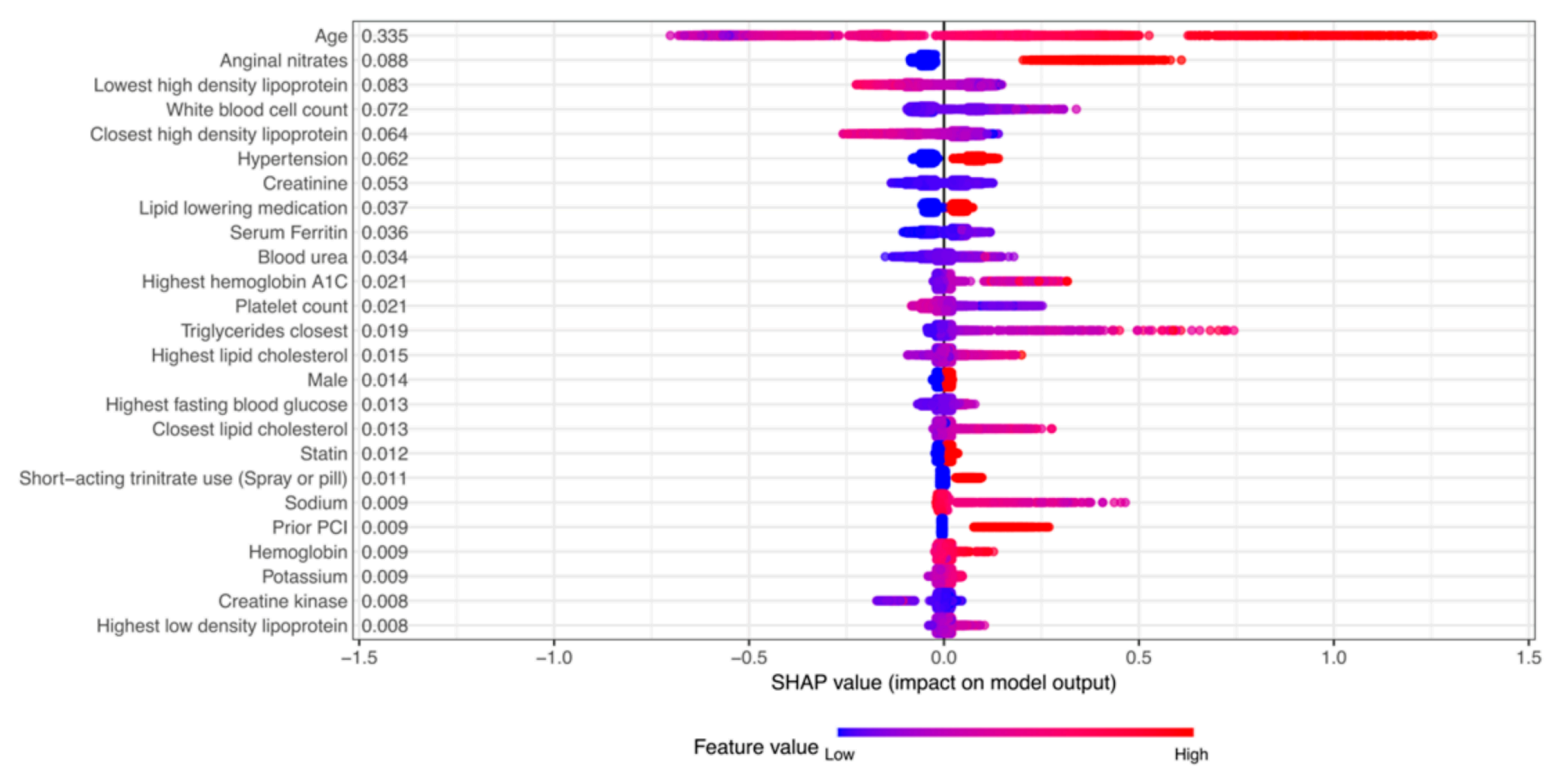
SHAP analysis of the internal cohort (Calgary). Each point in the plot represents one patient; horizontal position indicates the SHAP value, or contribution to the predicted probability of ICA referral, and colour encodes the feature value from low to high. Features are ranked by mean absolute SHAP value, shown to the left of each row. Age, anginal nitrate usage and lowest HDL cholesterol were the three highest-ranked predictors, matching the gain-based ranking in Figure 2.

**Figure 4:**
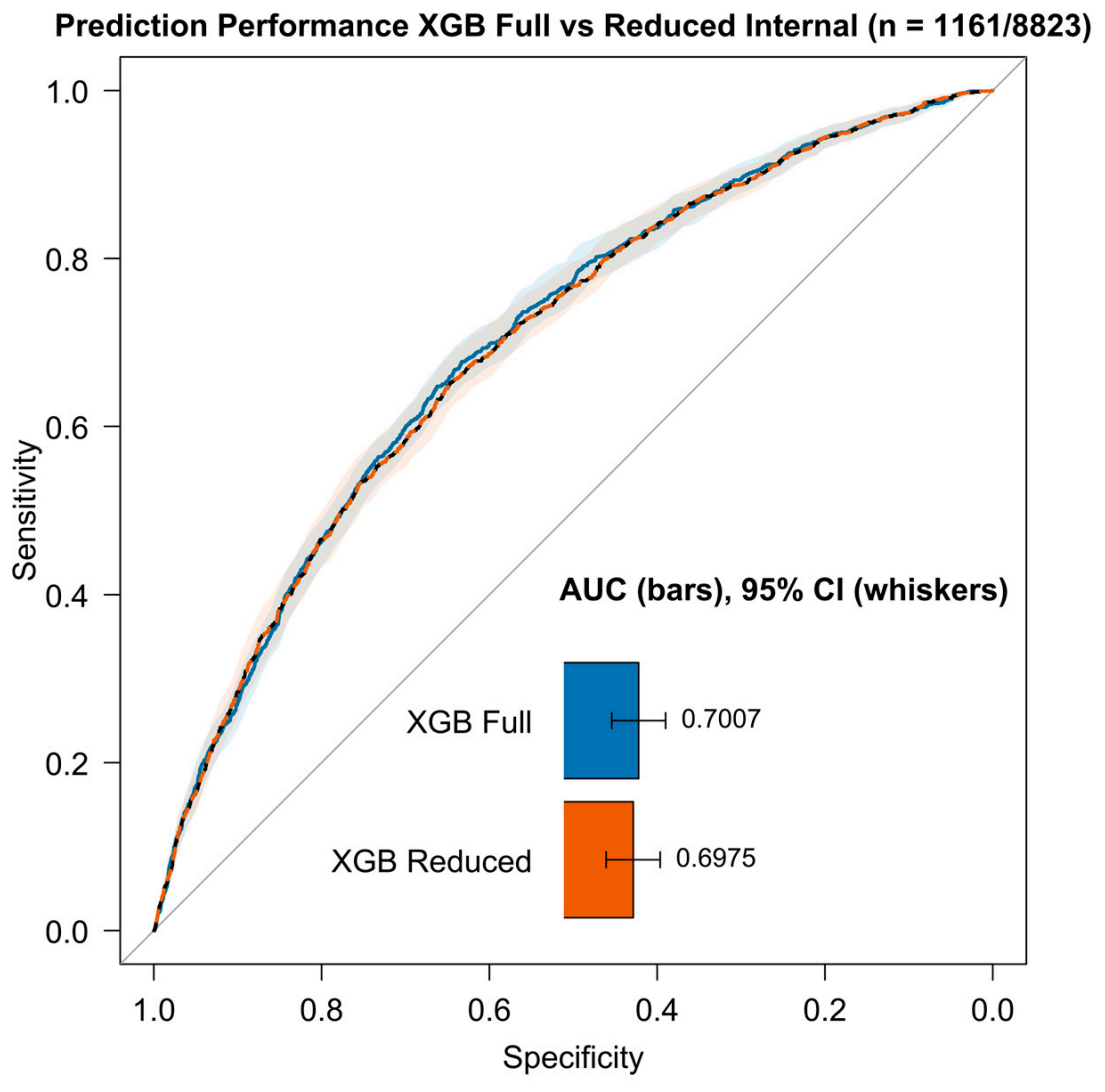
Discrimination of the full versus reduced XGBoost models in the internal cohort (Calgary). ROC curves for the full and reduced XGBoost models. Internal discrimination was statistically comparable (full AUC 0.701 versus reduced AUC 0.698; DeLong *p* = 0.16), indicating that feature reduction did not compromise discrimination in the development cohort. The diagonal denotes chance performance.

**Figure 5:**
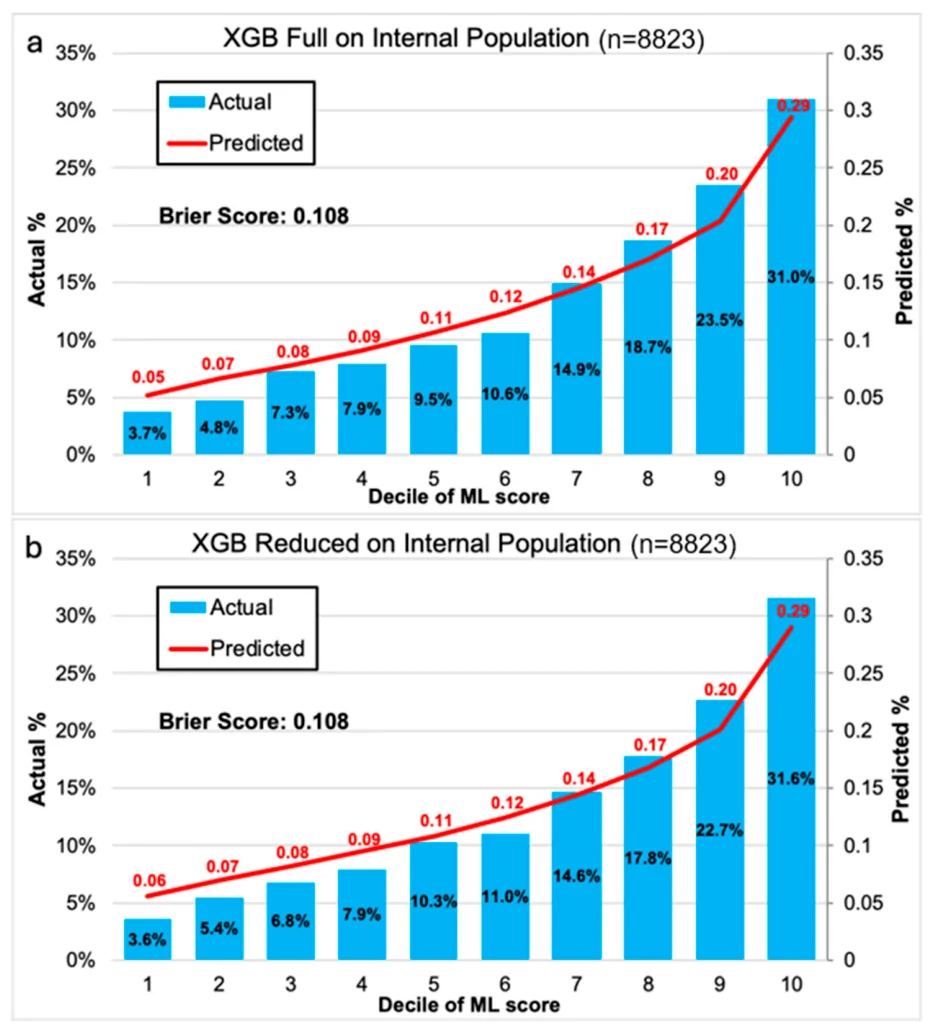
Calibration of the full and reduced XGBoost models in the internal cohort (Calgary). Calibration plots of predicted versus observed probability of ICA referral. (**a**) Full model (Brier 0.107; calibration slope 1.18, intercept −0.011). (**b**) Reduced 15-variable model (Brier 0.107; calibration slope 1.21, intercept −0.017). Both models showed good calibration-in-the-large, with slopes slightly above 1 indicating mild under-dispersion of predicted probabilities.

After gain-based feature importance and correlation filtering, we included 15 variables to train the model internally and test it externally on the reduced feature set. Gain-based feature importance analysis is visualized in Figure 2 and SHAP in Figure 3. Feature importance analysis revealed that age, anginal nitrates, lowest HDL, and closest HDL had the greatest contribution to predicting referral for ICA after CCTA. Internal performance for the full and reduced XGB models is shown in Figure 4. Our reduced model showed a similar AUC compared to the full model (0.698, 95% CI 0.681–0.713 vs. 0.701, 95% CI 0.685–0.717, *p* = 0.160). The reduced model had a similar F1 score (0.327 vs. 0.330, *p* = 0.37) and PPV (0.219 vs. 0.219, *p* = 0.996) compared to the full model. The full model had a greater NPV compared to the reduced model (0.928 vs. 0.925, *p* = 0.017), a higher sensitivity (0.677 vs. 0.652, *p* = 0.001), and lower specificity (0.633 vs. 0.647, *p* < 0.001). Table 2 shows a comprehensive comparison of model performance. Calibration stayed consistent between the full and reduced models, with the full model having a Brier score of 0.1076 compared to the reduced model’s Brier score of 0.1077 (*p* = 0.459) (see Figure 5). Both models had calibration intercepts near zero (full −0.011, 95% CI −0.074 to 0.052; reduced −0.017, −0.081 to 0.045) and calibration slopes were slightly above one (full 1.18, 95% CI 1.07 to 1.29; reduced 1.21, 1.10 to 1.33), indicating accurate calibration with mild under-dispersion of predicted risks (see Table 3).

**Figure 6:**
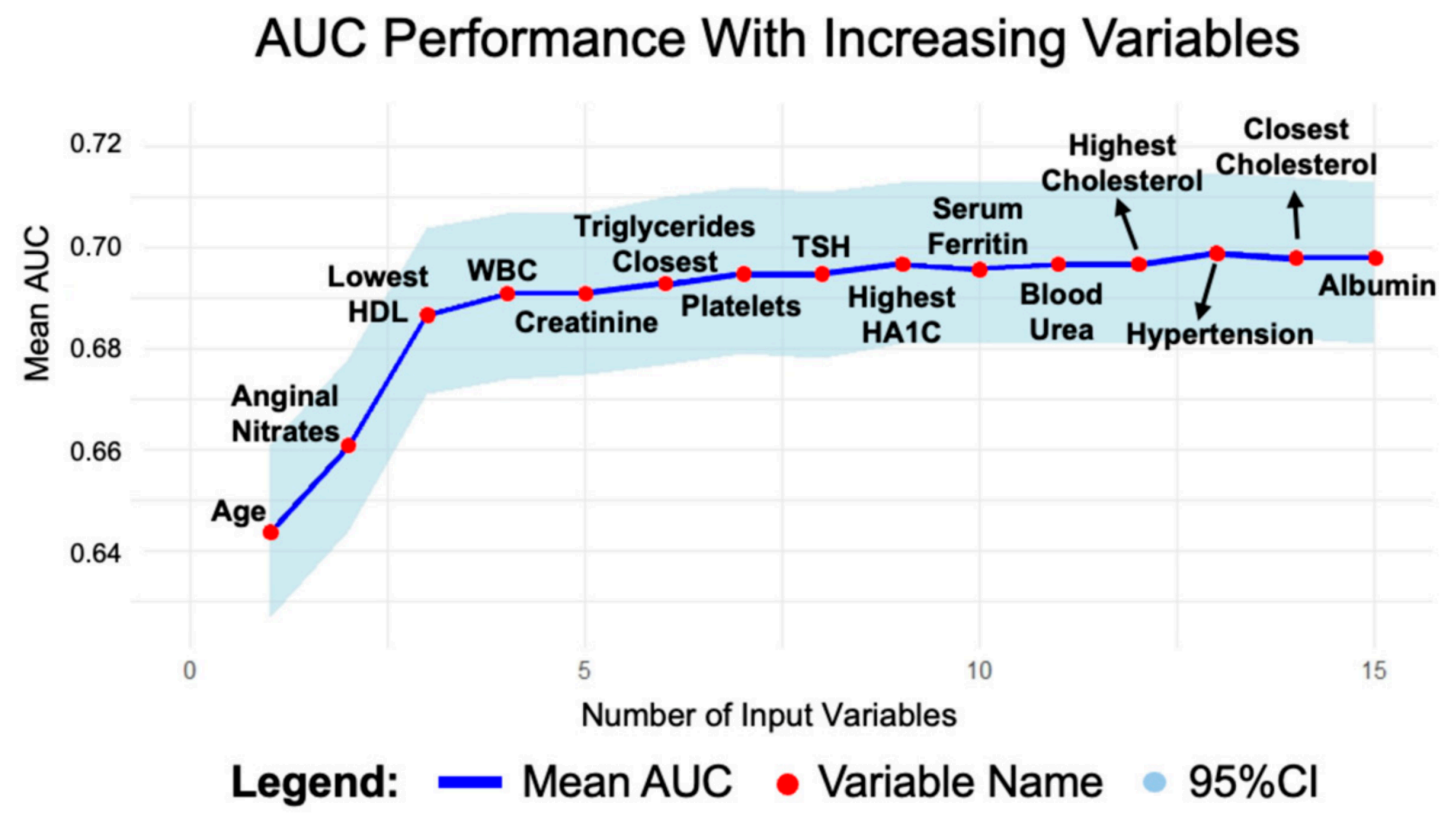
Sequential AUC progression in the internal cohort (Calgary). AUC is plotted as each top-ranking variable is added in descending order of gain importance. Performance plateaued after approximately 13 variables, reaching a maximum AUC of 0.699 (overall 0.698).

Results from our sequential model, made up of the top 15 variables from the gain-based feature importance after correlation filtering, are shown in Figure 6. This model achieved the highest AUC value of 0.699 after the addition of 13 variables. Internal results for the secondary external testing can be found in Figures S2 to S6.

**Figure 7:**
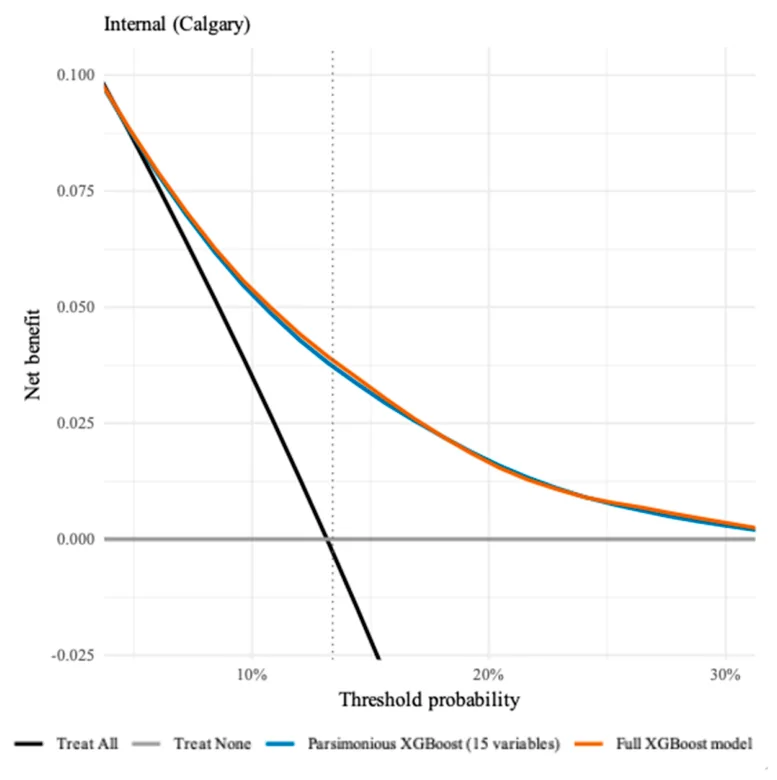
Decision curve analysis for the full and reduced XGBoost models in the internal cohort (Calgary). Internal cohort (Calgary, *n* = 8823). Net benefit is shown relative to referring all patients and referring no patients, across threshold probabilities from 5% to 30%. The dotted line indicates the Youden-derived threshold (0.134). The full and reduced models are nearly identical in the internal cohort.

Decision curves for the full and reduced models in the internal cohort are shown in Figure 7 and net benefit at selected thresholds in Table 4. Both models exceeded the net benefit of referring all patients and referring no patients across the thresholds examined, with the advantage over referring all becoming apparent above approximately 10% and diminishing above 20%. At the Youden-derived threshold applied (0.134), net benefit for the reduced model was 0.038, compared with −0.003 for referring all patients and zero for referring none, corresponding to a net reduction of 26.5 referrals per 100 patients relative to referring all without a reduction in true positives. Net benefit was 0.053 at a threshold of 10% and 0.031 at 15%, compared with 0.035 and −0.022 for referring all patients. At a threshold of 5% the model classified 98.2% of patients as positive, with a positive predictive value of 0.134, approximating the observed ICA rate. Net benefit of the two models differed by less than 0.002 at every threshold examined (0.038 vs. 0.039 at 0.134; 0.053 vs. 0.054 at 10%). Predicted probabilities exceeded 0.50 in 8 of 8823 patients (maximum 0.638). Classification consequences per 1000 patients are reported in Table S6. Decision curves across the full range of threshold probabilities (0–95%) for the internal cohort are shown in Figure S7.

### 3.3. External Results

**Figure 8:**
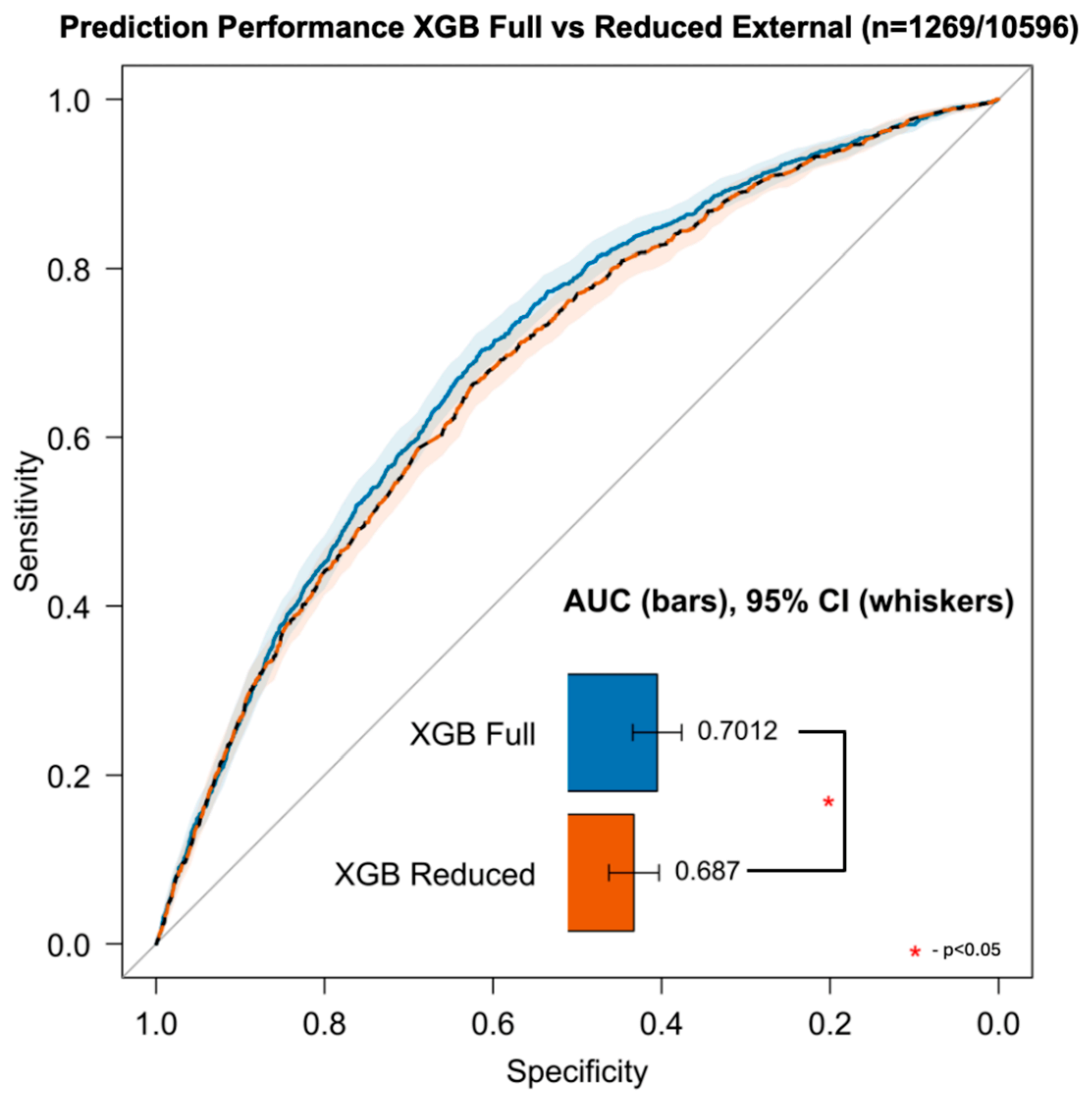
Discrimination of the full versus reduced XGBoost models in the external cohort (Edmonton). Receiver operating characteristic (ROC) curves for the full and reduced 15-variable models. External (Edmonton) cohort: the full model (AUC 0.701) modestly outperformed the reduced model (AUC 0.687; DeLong *p* < 0.001). The diagonal line denotes chance performance.

**Figure 9:**
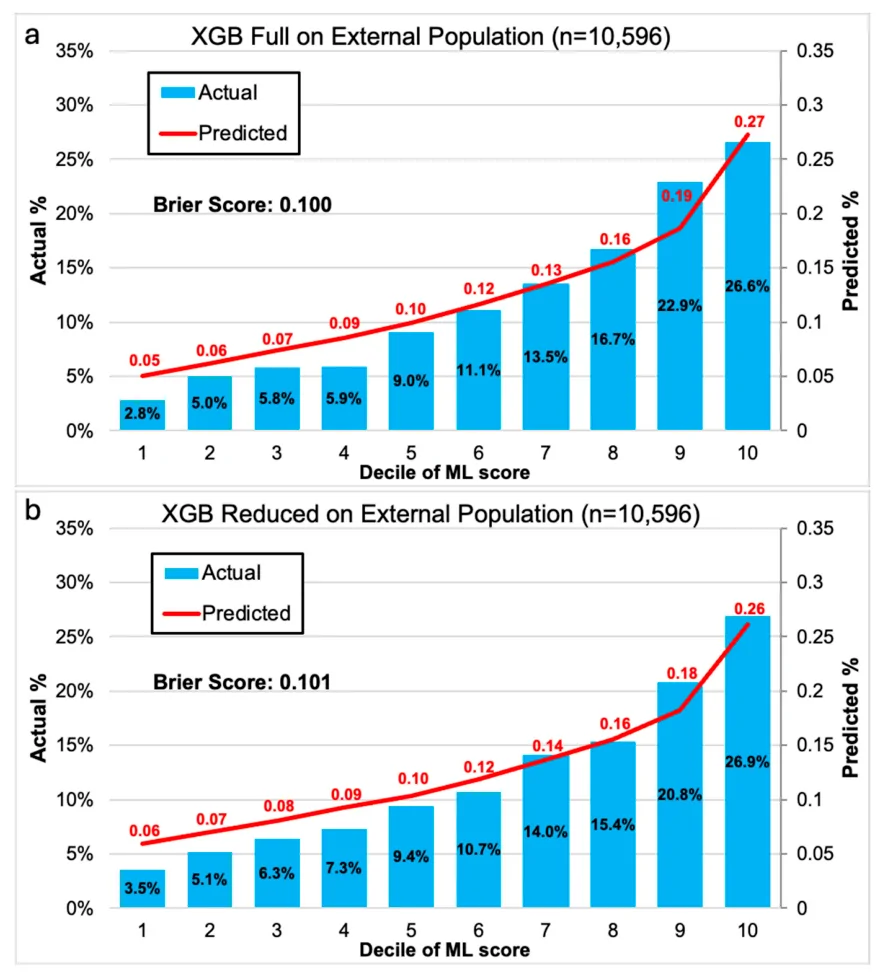
Calibration of the full and reduced XGBoost models in the external cohort (Edmonton). Calibration plots of predicted versus observed probability of ICA referral. (**a**) Full model (Brier 0.100; calibration slope 1.27, intercept −0.029). (**b**) Reduced 15-variable model (Brier 0.101; calibration slope 1.23, intercept −0.056).

**Figure 10:**
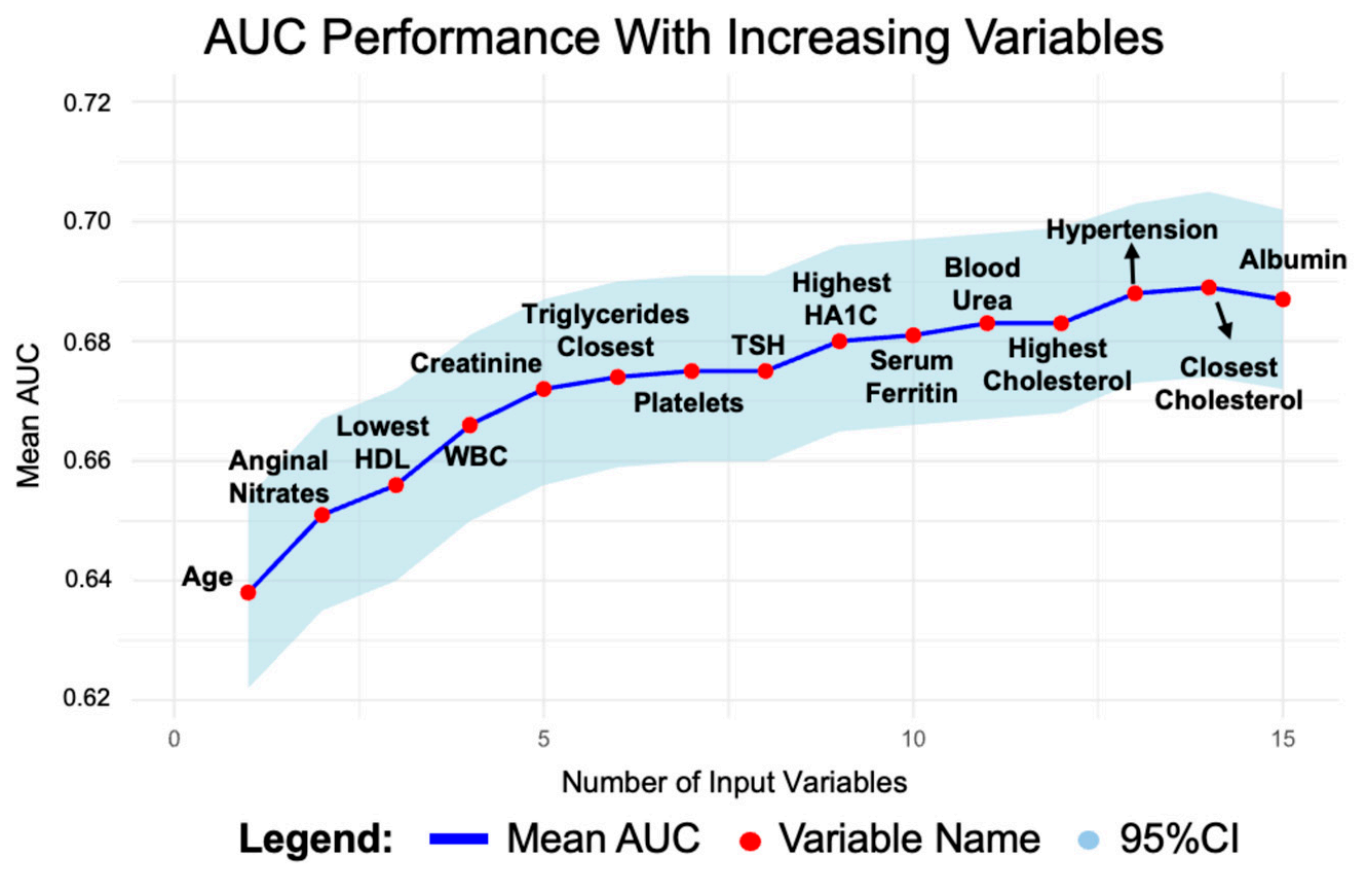
Sequential AUC progression in the external cohort (Edmonton). AUC is plotted as each top-ranked variable is added in descending order of gain importance. Performance plateaued after approximately 14 variables, reaching a maximum AUC of 0.689 (overall 0.687).

Results for prediction performance of the full and reduced XGB models in the external cohort are shown in Figure 8. The full model prior to reduction significantly outperformed the reduced-features model (AUC 0.701 vs. 0.687, *p* < 0.001). Compared to the full model, the reduced model achieved a lower F1 (0.310 vs. 0.296, *p* < 0.001), NPV (0.929 vs. 0.925, *p* < 0.05), PPV (0.207 vs. 0.196, *p* < 0.001), and specificity (0.680 vs. 0.666, *p* < 0.001) but a similar sensitivity (0.615 vs. 0.600, *p* = 0.0932). See Table 5 for a summary of performance differences between models. The full model had a significantly lower Brier score than the reduced model (full 0.100, reduced 0.101, *p* < 0.05), with calibration intercepts near zero (full −0.029, 95% CI −0.089 to 0.031; reduced −0.056, −0.116 to 0.003) and calibration slopes above one (full 1.27, 95% CI 1.16 to 1.39; reduced 1.23, 1.12 to 1.35) (see Figure 9 and Table 6). Our sequential model is shown in Figure 10. The sequential model achieved its highest AUC of 0.689 after the addition of 14 variables. SHAP analysis (see Figure S8) identified age as the dominant predictor of ICA referral after CCTA, with older age strongly increasing predicted referral probability, followed by anginal nitrate usage and lower HDL cholesterol. External results for secondary external testing can be found in Figures S9 to S12.

**Figure 11:**
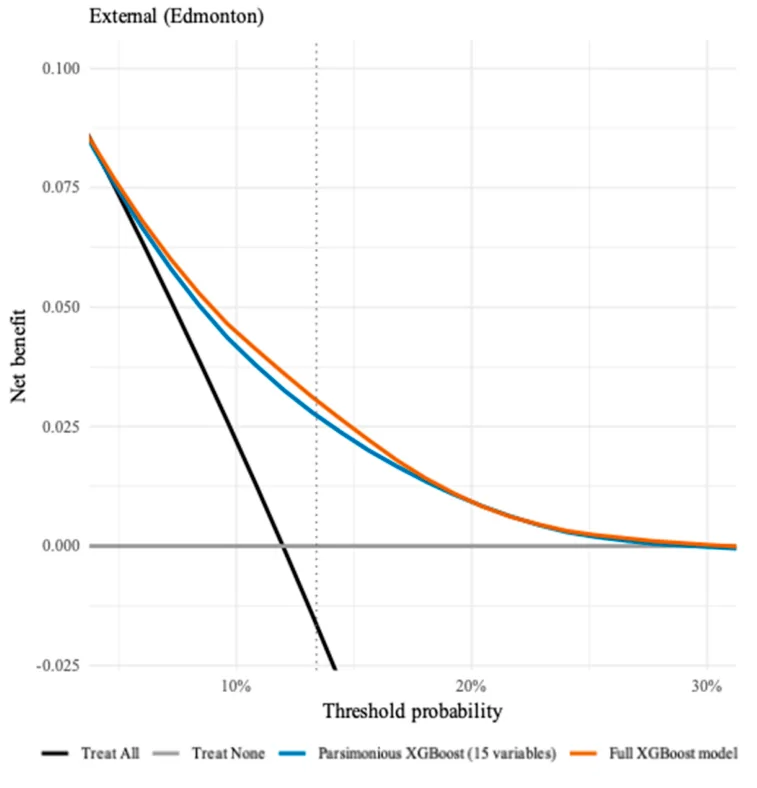
Decision curve analysis of the XGB full and XGB reduced models in the external cohort (Edmonton). External cohort (Edmonton, *n* = 10,596). Net benefit is shown relative to referring all patients and referring no patients across threshold probabilities from 5% to 30%. The dotted line indicates the Youden-derived threshold (0.134).

Decision curves for the external cohort are shown in Figure 11 with net benefit at selected thresholds in Table 7. At the Youden-derived threshold applied (0.134), net benefit for the reduced model was 0.026, compared with −0.016 for referring all patients and zero for referring none, corresponding to a net reduction of 27.5 referrals per 100 patients relative to referring all. Net benefit was 0.043 at a threshold of 10% and 0.021 at 15%, compared with 0.022 and −0.036 for referring all patients. At a threshold of 5% the model classified 99.0% of patients as positive, with a positive predictive value of 0.120. Net benefit was modestly lower for the reduced than the full model (0.026 vs. 0.030 at 0.134; 0.043 vs. 0.046 at 10%), consistent with the difference in external discrimination. Predicted probabilities exceeded 0.50 in 3 of 10,596 patients (maximum 0.562). Classification consequences per 1000 patients are reported in Table S6. Decision curves across the full range of threshold probabilities (0–95%) for the external cohort are shown in Figure S7.

## 4. Discussion

The primary objective of this study was to develop and externally validate a parsimonious XGB model capable of predicting ICA referral within 90 days of CCTA. Our reduced-features model achieved a comparable AUC to the full model during internal validation. However, in external testing, the reduced-features model had a lower prediction performance compared to the full model (0.687 vs. 0.701, *p* < 0.001). F1 score analysis revealed that the reduced model had a similar F1 score compared to the full model in internal training (0.327 vs. 0.330, *p* = 0.37) but was significantly lower in external testing (0.296 vs. 0.310, *p* < 0.001). Calibration based on the mean squared difference between actual and predicted probabilities was comparable in internal training but significantly different in external testing between the full and reduced models. The gain metric in the internal cohort identified age, anginal nitrates, and HDL cholesterol as the main drivers for ICA referral. Together these findings show that feature reduction preserved most of the full model’s predictive performance while substantially reducing the input burden, a prerequisite for, but not evidence of, clinical usefulness.

Models have not been extensively developed to predict referral to ICA following CCTA, which is a complex decision that incorporates pre-test information and CCTA results. The performance of the full XGB model is therefore a reflection of the complexity of predicting physician referral behaviour rather than a strictly defined pathological outcome. ICA referral decisions are shaped by clinical history, imaging findings, institutional practice patterns and patient-level factors. All these variables introduce variability that intrinsically limits the performance of prediction models [29,30]. Despite this variability, the consistent performance of the reduced model across both the internal and external cohorts is encouraging. The use of gain-based feature selection and correlation filtering allows for the effective elimination of redundant variables without sacrificing accuracy, yielding a model built on 15 features that preserved the predictive abilities of the full 156-variable model. Importantly, the sequential feature addition curve demonstrated that the AUC plateaued after approximately 13 variables internally and 14 variables externally, suggesting that a large proportion of the full feature set contributed less to the independent predictive value once the most informative variables were used.

The model does not incorporate CCTA findings, which are the principal determinants of downstream ICA [31]. It cannot replace clinical interpretation of the CCTA and is not intended to, and its moderate discrimination is consistent with this design. Clinical, laboratory and medication data available before CCTA capture only part of the information on which referral decisions are ultimately made. Its potential role lies earlier in the pathway, at the point of test selection, where estimating the probability of downstream invasive testing could inform whether CCTA or direct ICA is the more appropriate first test. Incorporating structured CCTA findings would be necessary for a model intended to support decisions after CCTA.

In the internal analysis, the full and reduced models had similar discrimination, indicating that the 15 variables that were retained captured essentially the same prediction signal as the full 156-variable model. Externally, the full model retained a small but significant advantage compared to the reduced model (AUC 0.701 vs. 0.687, *p* < 0.001). Discrimination was moderate in both cohorts in absolute terms, indicating that the models separate patients who proceed to ICA from those who do not with modest accuracy, and do not support use as a stand-alone determinant of referral. This significance should be interpreted in the context of the large external sample size (*n* = 10,596), in which even clinically negligible differences reach statistical significance [32]. An AUC difference of 0.014 is unlikely to change triage decisions at the point-of-care, and the reduced model was able to preserve the large majority of the full model’s prediction performance [33]. The same consideration applies to the differences in NPV (0.929 vs. 0.925) and specificity (0.680 vs. 0.666); although statistically significant in a cohort of 10,596 patients, differences of this magnitude would not alter the classification of a meaningful number of patients. Additionally, the difference in discrimination in external validation, in contrast to their performance internally, may be the result of differences in the magnitude and structure of predictor error between the internal and external cohorts. Variation in predictor measurement is known to affect the discriminative ability and transportability of models [34]. Since our parsimonious model relies on a smaller predictor set, the model is more sensitive to between-site variation because it has fewer correlated predictors to compensate when one is less complete in the external cohort [34]. This idea is consistent with and supported by the data in Table S3, where some of the reduced model’s predictors had a higher percentage of missingness externally than internally. Large differences in missingness between sites may limit the model performance and achievable AUC value, which in turn limits the reliability of the model when generalizing to external datasets. Therefore, it is plausible that the reduced model in external validation may have performed similarly to the internal model if the external cohort resembled the internal cohort more closely.

Calibration was assessed using Brier scores, calibration intercept and slope. Brier scores were nearly identical for both models internally and externally, 0.107 and 0.101 respectively. However, considering the significant difference externally between the full and reduced models based on squared difference, Brier score must be interpreted considering the low event rate in each cohort, as it is influenced by outcome prevalence. Interpreting calibration using intercept and slope arguably led to a more informative assessment of the calibration between models. In the internal and external testing cohorts both the full and reduced models achieved an intercept close to zero and a 95% CI that overlapped 0, indicating a good calibration-in-the-large in both cohorts and models [35]. Calibration slopes for both models in both cohorts were slightly above one, with 95% CIs that excluded 1, indicating that the predicted probabilities were marginally too moderate rather than extreme [36]. This is the opposite of the overfitting typically seen in clinical prediction models when they are exposed to new data, which produces slopes below 1 [37]. Calibration metrics found through our models represent a conservative form of miscalibration that could be corrected using logistic recalibration before deployment [37]. Comparable Brier scores, slopes and intercepts between the full and reduced models demonstrate that parsimony did not compromise the model’s risk estimates. Preservation of model calibration, despite differences in case mix and event rates between populations, supports the generalizability of the reduced model’s probability estimates. Calibration was, however, assessed in cohorts with similar event rates. Downstream ICA rates were 13.2% in Calgary and 12.0% in Edmonton, but only 3.0% in Ottawa. Differences in case mix contribute (Table S5), but these differences are more consistent with institutional variation in referral thresholds and access to catheterization, where population rates of cardiac catheterization vary substantially across Canadian health regions [38]. Due to the outcome reflecting referral practice, our model mirrors the baseline referral rate of its development site. Therefore, recalibration of the intercept to the local ICA rate, and selection of a locally appropriate threshold, would be required before use at any new site

Gain-based feature importance analysis identified age, anginal nitrates, and HDL cholesterol as the three strongest predictors for ICA referral following CCTA, with white blood cell count, creatinine, and triglycerides also being high contributors. The prominence of age aligns with clinical evidence that older patients carry a higher pre-test probability of obstructive disease and are more likely to be referred for invasive testing [9].

Patient usage of anginal nitrates likely reflects their role as a clinical indicator for active or reoccurring anginal symptoms. Nitrate use, including short-acting nitroglycerine and long-acting nitrates, has been recommended by international guidelines for symptomatic relief in patients with chronic coronary syndromes [39]. Patients on this medication typically have an established diagnosis or ongoing symptoms related to coronary disease, thus increasing the probability of those patients being triaged for downstream invasive testing. Medication features like anginal nitrates may function as clinical indicators of physician-interpreted disease severity, which may not be captured in administrative data.

The importance of HDL and triglycerides is consistent with their roles as established biomarkers for dyslipidemia and cardiovascular risk, and their presence as one of the higher predictors suggests that lipid burden prior to or at the time of CCTA informs downstream referral decisions [14,15].

Serum creatinine and white blood cell count represent two non-traditional cardiovascular risk pathways. Creatinine serves as a dual indicator of vascular and metabolic risk due to its use in assessing renal function. Patients with decreased renal functioning are independently associated with increased coronary plaque burden, calcification, and worse cardiovascular outcomes [40]. Renal function is also relevant to the downstream consequences of testing. Each procedure in a CCTA-then-ICA pathway adds radiation exposure and iodine contrast, where ICA carries additional procedural risk [6]. In the DISCHARGE trial, major procedure-related complications occurred in 1.9% of patients assigned to initial ICA compared with 0.5% assigned to initial CCTA [5]. Repeated contrast administration is of particular concern in patients with impaired renal function, where contrast-associated acute kidney injury (CA-AKI) is more common [41]. Avoiding unnecessary sequential testing is therefore of value in this cohort of patients.

Increased white blood cell count is a non-specific marker for inflammation and has been associated with adverse cardiovascular outcomes and the presence and severity of CAD [42]. This association is consistent with the physiological process inflammation has in the development of atherosclerotic plaque, and with the broader clinical recognition of inflammation as a cardiovascular risk pathway [43]. Taken together, the feature importance profile of the XGB model suggests that the model draws from established cardiovascular risk markers and functional indicators, rather than demographic or administrative variables alone.

Two important metrics were utilized in this work: gain-based importance to rank and select features during model development and SHAP to interpret model behaviour. Comparing the two methods using the internal cohort showed that age, anginal nitrates and lowest HDL were the top three predictors in both methods, and the same five variables occupied the five highest positions. The two methods showed divergence within the mid-ranked variables and followed a pattern where binary indicators were ranked consistently higher by SHAP, and continuous variables were ranked higher by gain. This is consistent with the observation that gain-based feature selection has bias toward continuous and high-cardinality variables, and supports the use of SHAP as a complementary interpretive tool rather than a replacement for gain [44].

Direct comparison to prior work is challenging, as there is no existing model specifically designed to predict ICA referral following CCTA. Most traditional risk scores, such as the Diamond–Forrester and CAD Consortium models, aim to estimate the likelihood of obstructive CAD rather than downstream testing decisions [9,45]. Similarly, prior ML studies in cardiac imaging have mainly focused on anatomic disease detection or MACE prediction, tasks that fundamentally differ from ours in terms of modelling physician behaviour [46,47,48]. In the broader scope of the cardiovascular ML literature, XGB-based models have reported AUC values in the range of 0.73 to 0.93 for CAD-related outcomes [49,50,51]. Our observed AUCs are slightly lower, which is expected and consistent with the greater difficulty of predicting physician behaviour compared to pathological endpoints. Furthermore, the application of systematic feature reduction to cardiovascular prediction models remains underexplored in the available literature. Verma et al. evaluated cardiac surgery outcomes with a parsimonious ML model using only preoperative variables, which achieved strong discrimination for the outcome [52]. Awan et al. showed that feature reduction from 47 to 8 predictors preserved AUC in heart failure readmission prediction [53]. Our findings extend these studies to the novel context of post-CCTA ICA triage, showing that principled feature reduction is feasible without meaningful performance loss.

We defined the intended decision as whether a patient being considered for CCTA should instead proceed directly to ICA, staying with the guideline pathway for patients with very high clinical likelihood [3]. Under this decision, a false-positive classification directs a patient to an invasive procedure they would not otherwise have undergone, with its associated procedural risk, contrast exposure and radiation. A false-negative classification results in CCTA followed by ICA, which is the current pathway. Due to the harm of an unnecessary invasive procedure exceeding that of an additional non-invasive test, the threshold probabilities relevant to this decision lie at the higher end of the range, above approximately 50%.

Predicted probabilities in our cohorts rarely reached this range: 8 of 8823 internal and 3 of 10,596 external patients had a predicted probability above 0.50. This was expected for a well-calibrated model in a population with a large class imbalance (13.2% and 12.0%) and predictions clustered above 50% would instead suggest overconfidence. The model therefore does not identify a group of patients in whom CCTA could be bypassed in favour of direct ICA. Where the model did add net benefit, at threshold probabilities of approximately 10% to 30%, the implied harm ratio corresponds to treating one missed referral as equivalent to between nine and two unnecessary referrals. This trade-off is defensible for prioritizing review or scheduling but not for selecting patients for an invasive procedure. Taken together, these analyses indicate that the model’s current clinical role, if any, lies in risk stratification at the point of test selection rather than in directing individual patients to invasive assessment.

There are many notable strengths to our study. First, this is, to our knowledge, the first reduced-features model specifically designed to predict ICA referral following CCTA, addressing a gap in the prediction of downstream invasive testing. Secondly, the use of a large, multi-site Canadian cohort with external validation reduced the risk of model overfitting and supports the ability of the ML models to generalize across geographically and institutionally distinct patient populations, an attribute that remains unusual in cardiovascular ML analysis [54]. Third, the transparent feature reduction strategy, using gain-based feature reduction, pairwise correlation filtering and sequential feature addition, provides a reproducible and interpretable path from a large administrative dataset to a parsimonious model [55]. Finally, the integration of SHAP-based explainability increases clinician trust by providing variable rationale for individual predictions, addressing a barrier to ML adoption in healthcare [24].

A few limitations of this study warrant acknowledgment. First, the retrospective cohort design introduces potential selection bias, and referral decisions captured in the administrative data may not fully reflect the clinical reasoning that drove them. Second, there was a significant class imbalance in both internal (13.2%) and external (12%) cohorts, meaning that the models were trained on a population where subsequent ICAs represented a small minority of cases. Imbalance of this size can reduce PPV, inflate specificity, and limit the model’s sensitivity to true positive cases [56]. Third, the outcome in this study reflects clinical practice rather than a pathological endpoint. A model trained on historical referrals learns the patterns of existing practice, including referrals that would not meet appropriateness criteria; in a large national registry, obstructive CAD was found in only about 38% of patients undergoing elective ICA [57]. Our model therefore estimates the probability that a patient will proceed to ICA under current practice, not whether ICA is warranted, and could reproduce existing over- or under-referral if applied without further validation [30]. Evaluating the model against angiographic endpoints, such as obstructive CAD or revascularization, using linked APPROACH registry data is a priority for future work. Fourth, the 15 variables were selected using gain-based importance from a single model family. Importance rankings can differ between algorithms and an alternative approach would be to compare SHAP-derived rankings across several model families to identify predictors that consistently contribute towards prediction regardless of the learner. This method was not explored in this research, and therefore, the retained feature set may reflect the bias of gradient-boosted trees. Because ranking was pooled across folds rather than re-derived within each fold, the retained feature set was also informed by the whole internal cohort, and internal estimates of performance may be modestly optimistic. The external cohorts were held out during feature ranking, correlation filtering, sequential feature addition and hyperparameter tuning, so external estimates are unaffected. Lastly, several predictor variables were derived from provincial administrative databases specific to Alberta and Ontario healthcare systems, including PIN and Alberta Laboratory Services. The availability of equivalent administrative data infrastructure may vary across jurisdictions, which could limit the direct portability of this model without comparable data structures. Laboratory values were also determined using a 3-year lookback and, although the measurement closest to the index CCTA was used preferentially, some values may not reflect the patient’s status at the index examination. A sensitivity analysis restricted to more recent measurements was not undertaken and would be informative in future work.

## 5. Conclusions

Our study presents a parsimonious, externally tested XGB model for predicting ICA, built with a reduced and clinically accessible feature set. The reduced model achieved strong and comparable prediction and calibration performance across the internal cohort and small but significantly lower performance externally, with gain-based feature importance supporting its clinical interpretability. Clinical deployment would require demonstration that the model improves appropriate selection of patients for ICA, rather than reproducing historic referral behaviour, ideally in a prospective evaluation with locally recalibrated thresholds. Future work related to the reduced model will focus on utilizing and comparing various feature selection methods against gain-based feature importance, including LASSO regression and SHAP rankings aggregated across multiple models. Additionally, we will focus on sex-stratified ML models to further assess integration into point-of-care clinical workflows as a decision support tool to fairly evaluate the sex differences in CAD presentation and ICA referral.

## Figures and Tables

**Table 1:** Baseline characteristics of the internal (Calgary) and external (Edmonton) cohorts. Demographics, medical history, medications, and laboratory values are summarized for each cohort with between-cohort comparison.

Variable	Calgary (*n* = 8823)	Edmonton (*n* = 10,596)	*p*-Value
Male, *n* (%)	5084 (57.6%)	5730 (54.1%)	<0.001
Age, median (IQR)	59 (49–66)	57 (49–65)	<0.001
Subsequent ICA, *n* (%)	1161 (13.2%)	1269 (12.0%)	0.014
Medical History, *n* (%)			
Prior CAD	1038 (11.8%)	846 (8.0%)	<0.001
Diabetes	2031 (23.0%)	2542 (24.0%)	0.116
Hypertension	3183 (36.1%)	3793 (35.8%)	0.697
Dyslipidemia	6667 (75.6%)	6359 (60.0%)	<0.001
COPD	327 (3.7%)	531 (5.0%)	<0.001
Prior PCI	253 (2.9%)	201 (1.9%)	<0.001
Cancer	812 (9.2%)	619 (5.8%)	<0.001
Medications, *n* (%)			
Anginal Nitrates	902 (10.2%)	649 (6.1%)	<0.001
Beta Blocker	5628 (63.8%)	6378 (60.2%)	<0.001
Anticoagulant	695 (7.9%)	589 (5.6%)	<0.001
Labs, median (IQR)			
Lowest HDL	1.18 (0.96–1.45)	1.20 (1.01–1.45)	<0.001
Triglycerides Closest	1.39 (0.98–2.02)	1.49 (1.06–2.11)	<0.001
White Blood Cells	6.5 (5.4–7.8)	6.7 (5.6–8.2)	<0.001

CAD, coronary artery disease; IQR, interquartile range; COPD, chronic obstructive pulmonary disease; PCI, percutaneous coronary intervention; HDL, high-density lipoprotein; ICA, invasive coronary angiography.

**Table 2:** Statistical metrics comparing XGB full and XGB reduced in the internal (Calgary) cohort.

Metric	XGB Full	XGB Reduced	Δ (95% CI)	*p*-Value
ROC AUC	0.701	0.698	0.0032 (−0.0013 to 0.0077)	0.16
Sensitivity	0.677	0.652	0.0251 (0.0107 to 0.0400)	0.001
Specificity	0.633	0.647	−0.0136 (−0.0194 to −0.0076)	7.38 × 10^−6^
PPV	0.219	0.219	0.0000 (−0.0046 to 0.0047)	0.996
NPV	0.928	0.925	0.0037 (0.0007 to 0.0067)	0.017
F1 score	0.330	0.327	0.0031 (−0.0036 to 0.0099)	0.37

Δ denotes the difference between the full and reduced XGBoost models (full minus reduced). AUC, area under the receiver operating characteristic curve; PPV, positive predictive value; NPV, negative predictive value; XGB, extreme gradient boosting.

**Table 3:** Calibration performance of the XGB full and XGB reduced models in the internal (Calgary) cohort.

Model	Brier Score	Calibration Intercept (95% CI)	Calibration Slope (95% CI)
XGB Full	0.1076	−0.011 (−0.074 to 0.052)	1.178 (1.072 to 1.285)
XGB Reduced	0.1077	−0.017 (−0.081 to 0.045)	1.214 (1.104 to 1.325)

Lower Brier scores indicate better overall calibration. A calibration intercept of 0 indicates no systematic over- or under-estimation of risk (values > 0 indicate underestimation); a calibration slope of 1 indicates ideal agreement between predicted and observed risk (values < 1 indicate probability compression/overfitting).

**Table 4:** Net benefit of the XGB full and XGB reduced models in the internal cohort (Calgary).

Threshold Probability	Refer All	Refer None	XGB Full	XGB Reduced	Referrals Avoided Per 100 (Reduced)
5.0%	0.0859	0.0000	0.0872	0.0866	—
10.0%	0.0351	0.0000	0.0535	0.0529	16.0
13.4%	−0.0028	0.0000	0.0394	0.0382	26.5
15.0%	−0.0217	0.0000	0.0325	0.0312	30.0
20.0%	−0.0855	0.0000	0.0160	0.0173	41.1
30.0%	−0.2410	0.0000	0.0034	0.0026	—

Net benefit is expressed as net true positives per patient. Referring none has a net benefit of zero at every threshold by definition. A threshold probability of 0.134 is the Youden-derived threshold applied. Referrals avoided per 100 patients is reported between 10% and 20%, the range over which the model provides a clear advantage over both default strategies. XGB, extreme gradient boosting.

**Table 5:** Statistical metrics comparing XGB full and XGB reduced for external testing (Edmonton).

Metric	XGB Full	XGB Reduced	Δ (95% CI)	*p*-Value
ROC AUC	0.701	0.687	0.014 (0.010 to 0.019)	6.81 × 10^−10^
Sensitivity	0.615	0.600	0.015 (−0.002 to 0.032)	0.0932
Specificity	0.680	0.666	0.014 (0.008 to 0.019)	3.16 × 10^−6^
PPV	0.207	0.196	0.011 (0.006 to 0.016)	6.52 × 10^−5^
NPV	0.929	0.925	0.004 (0.001 to 0.007)	0.0087
F1 score	0.310	0.296	0.014 (0.006 to 0.022)	4.31 × 10^−4^

Δ denotes the difference between the full and reduced XGBoost models (full minus reduced). AUC, area under the receiver operating characteristic curve; PPV, positive predictive value; NPV, negative predictive value; XGB, extreme gradient boosting.

**Table 6:** Calibration performance of the XGB full and XGB reduced models in the external (Edmonton) cohort.

Model	Brier Score	Calibration Intercept (95% CI)	Calibration Slope (95% CI)
XGB Full	0.1002	−0.029 (−0.089 to 0.031)	1.274 (1.163 to 1.385)
XGB Reduced	0.1008	−0.056 (−0.116 to 0.003)	1.232 (1.119 to 1.346)

Lower Brier scores indicate better overall calibration. A calibration intercept of 0 indicates no systematic over- or under-estimation of risk (values > 0 indicate underestimation); a calibration slope of 1 indicates ideal agreement between predicted and observed risk (values < 1 indicate probability compression/overfitting).

**Table 7:** Net benefit of the XGB full and XGB reduced models in the external cohort (Calgary).

Threshold Probability	Refer All	Refer None	XGB Full	XGB Reduced	Referrals Avoided Per 100 (Reduced)
5.0%	0.0734	0.0000	0.0738	0.0734	—
10.0%	0.0220	0.0000	0.0460	0.0429	18.8
13.4%	−0.0164	0.0000	0.0299	0.0261	27.5
15.0%	−0.0356	0.0000	0.0243	0.0211	32.1
20.0%	−0.1000	0.0000	0.0086	0.0091	43.8
30.0%	−0.2570	0.0000	0.0000	0.0001	—

Net benefit is expressed as net true positives per patient. Referring none has a net benefit of zero at every threshold by definition. A threshold probability of 0.134 is the Youden-derived threshold applied, derived internally and applied without re-tuning. Referrals avoided per 100 patients is reported between 10% and 20%. XGB—extreme gradient boosting.

## Data Availability

The data supporting this analysis can be made available upon written request and after appropriate data sharing agreements are in place.

## Supplementary Materials

The following supporting information can be downloaded at: https://www.mdpi.com/article/10.3390/jcm15197641/s1, Table S1: Predictor domains used in ML models to train internal models; Table S2: Summary of dataset sources used to obtain data for internal and external cohorts; Table S3: Values with missingness in the internal and external cohorts; Table S4: Missingness in the Calgary (internal) and Ottawa (external) cohorts for secondary testing; Table S5: Population characteristics, Calgary and Ottawa; Table S6: Classification consequences of the reduced XGBoost model per 1000 patients at selected threshold probabilities; Figure S1: Correlation heatmap for the internal cohort (Calgary); Figure S2: Correlation heatmap for the internal cohort (Calgary) in secondary testing; Figure S3: Gain-based feature importance for internal training (Calgary) in secondary testing; Figure S4: Prediction performance of the full and reduced XGBoost models on the internal cohort (Calgary) in secondary testing; Figure S5: Model calibration for the full and reduced XGBoost models on the internal cohort (Calgary) in secondary testing; Figure S6: Sequential AUC progression in the internal cohort (Calgary) in secondary testing; Figure S7: Decision curve analysis across the full range of threshold probabilities (0–95%) for the internal (Calgary) and external (Edmonton) cohorts; Figure S8: SHAP Beeswarm plot showing feature rank and directional SHAP values for the top features in the external cohort (Edmonton); Figure S9: SHAP beeswarm plot for the external cohort (Ottawa) in secondary testing; Figure S10: Prediction performance of the full and reduced XGBoost models on the external cohort (Ottawa) in secondary testing; Figure S11: Model calibration for the full and reduced XGBoost models on the external cohort (Ottawa) in secondary testing; Figure S12: Sequential AUC progression in the external (Ottawa) cohort for secondary testing.

## Abbreviations

The following abbreviations are used in this manuscript:

AUC	Area under the receiver operating characteristic curve
CAD	Coronary artery disease
CA-AKI	Contrast-associated acute kidney injury
CCTA	Coronary computed tomography angiography
CI	Confidence interval
HDL	High-density lipoprotein
ICA	Invasive coronary angiography
IQR	Interquartile range
ML	Machine learning
NPV	Negative predictive value
PIN	Pharmaceutical Information Network
PPV	Positive predictive value
ROC	Receiver operating characteristic
SHAP	SHapley Additive exPlanations
XGB	Extreme gradient boosting
