# Supplementary material for: A Parsimonious Machine Learning Model for Predicting Invasive Coronary Angiography Referral After Coronary Computed Tomography Angiography: Development and Multicentre External Validation
Source: J Clin Med. 2026 Oct 2;15(19):7641. doi: 10.3390/jcm15197641 (PMC13665986; doi:10.3390/jcm15197641)
Supplement: Supplementary file 1 [file jcm-15-07641-s001.zip › jcm-4557178-supplementary.pdf]

JCM_alec_chunta

JCM supplementary material:

Table S1: Predictor domains used in ML models to train internal models

Variable	Category	Description
Age	Demographics	Age in years
Sex	Demographics	Female – 0, Male – 1
Hemoglobin	Labs	Closest hemoglobin within 3 years prior to date of index visit
Hematocrit	Labs	Closest hematocrit within 3 years prior to date of index visit
Red cell distribution width	Labs	Closest red cell distribution width (RDW) within 3 years prior to date of index visit
White blood cell count	Labs	Closest white cell count within 3 years prior to date of index visit
Platelet count	Labs	Closest platelet count within 3 years prior to date of index visit
International normalized ratio	Labs	Closest INR within 3 years prior to date of index visit
Partial thromboplastin time	Labs	Closest partial thromboplastin time (PTT) within 3 years prior to date of index visit
Albumin	Labs	Closest albumin within 3 years prior to date of index visit
Alkaline phosphatase	Labs	Closest alkaline phosphatase within 3 years prior to date of index visit
Alanine transaminase	Labs	Closest alanine transaminase (ALT) within 3 years prior to date of index visit
Aspartate transaminase	Labs	Closest aspartate transaminase (AST) within 3 years prior to date of index visit
Total bilirubin	Labs	Closest total bilirubin within 3 years prior to date of index visit
Direct bilirubin	Labs	Closest direct bilirubin within 3 years prior to date of index visit
Indirect bilirubin	Labs	Closest indirect bilirubin within 3 years prior to date of index visit
Blood urea	Labs	Closest blood urea within 3 years prior to date of index visit
Creatinine	Labs	Closest serum creatinine within 3 years prior to date of index visit

Urinary albumin/Cr ratio	Labs	Closest urinary album creatinine ratio within 3 years prior to date of index visit
Sodium	Labs	Closest serum sodium within 3 years prior to date of index visit
Potassium	Labs	Closest serum potassium within 3 years prior to date of index visit
Chloride	Labs	Closest serum chloride within 3 years prior to date of index visit
Creatine kinase	Labs	Closest total CK within 3 years prior to date of index visit
Troponin - closest	Labs	Closest troponin I within 3 years prior to date of index visit
Troponin - highest	Labs	Highest troponin I within 3 years prior to date of index visit
Closest high sensitivity Troponin T level	Labs	Closest high-sensitivity troponin T within 3 years prior to date of index visit
Highest high sensitivity troponin T level	Labs	Highest high-sensitivity troponin T within 3 years prior to date of index visit
High sensitivity C reactive protein	Labs	Closest high-sensitivity C-reactive protein within 3 years prior to date of index visit
Erythrocyte sedimentation rate	Labs	Closest ESR within 3 years prior to date of index visit
Closest NT-Pro-BNP level	Labs	Closest NT-proBNP within 3 years prior to date of index visit
Highest NT-Pro-BNP level	Labs	Highest NT-proBNP within 3 years prior to date of index visit
Thyroid stimulating hormone	Labs	Closest TSH within 3 years prior to date of index visit
Closest fasting blood glucose	Labs	Closest fasting blood glucose within 3 years prior to date of index visit
Highest fasting blood glucose	Labs	Highest fasting blood glucose within 3 years prior to date of index visit
Closest random blood glucose	Labs	Closest random blood glucose within 3 years prior to date of index visit
Highest random blood glucose	Labs	Highest random blood glucose within 3 years prior to date of index visit
Closest post prandial blood glucose	Labs	Closest 2-hours post-prandial blood glucose within 3 years prior to date of index visit

Highest post prandial blood glucose	Labs	Highest 2-hours post-prandial blood glucose within 3 years prior to date of index visit
Closest hemoglobin A1C	Labs	Closest HbA1C within 3 years prior to date of index visit
Highest hemoglobin A1C	Labs	Highest HbA1C within 3 years prior to date of index visit
Closest lipid cholesterol	Labs	Closest total cholesterol within 3 years prior to date of index visit
Highest lipid cholesterol	Labs	Highest total cholesterol within 3 years prior to date of index visit
Closest low-density lipoprotein	Labs	Closest LDL cholesterol within 3 years prior to date of index visit
Highest low-density lipoprotein	Labs	Highest LDL cholesterol within 3 years prior to date of index visit
Closest high-density lipoprotein	Labs	Closest HDL cholesterol within 3 years prior to date of index visit
Lowest high-density lipoprotein	Labs	Lowest HDL cholesterol within 3 years prior to date of index visit
Triglycerides closest	Labs	Closest serum triglycerides within 3 years prior to date of index visit
Triglycerides highest	Labs	Highest serum triglycerides within 3 years prior to date of index visit
Blood iron	Labs	Closest serum iron within 3 years prior to date of index visit
Total iron binding capacity	Labs	Closest total iron binding capacity (TIBC) within 3 years prior to date of index visit
Serum ferritin	Labs	Closest serum ferritin within 3 years prior to date of index visit
Non-ASA anti platelet	Medications	Non-aspirin anti-platelet medication use (from PIN database) within 180 days prior to date of index visit
Anti-platelet ASA agent	Medications	Aspirin use (from PIN database) within 180 days prior to date of index visit
Oral anticoagulant use	Medications	Oral anti-coagulant medication use (from PIN database) within 180 days prior to date of index visit

Anginal nitrates	Medications	Nitrate use (from PIN database) within 180 days prior to date of index visit
Long-acting trinitrate use (Patch)	Medications	Long-acting (patch or ointment) trinitrate use (from PIN database) within 180 days prior to date of index visit
Short-acting trinitrate use (spray or pill)	Medications	Short-acting (sub-lingual spray or pill) trinitrate use (from PIN database) within 180 days prior to date of index visit
Isosorbide mononitrate	Medications	Mononitrate medication use (from PIN database) within 180 days prior to date of index visit
Isosorbide dinitrate	Medications	Dinitrate medication use (from PIN database) within 180 days prior to date of index visit
Ranolazine	Medications	Ranolazine medication use (from PIN database) within 180 days prior to date of index visit
ARB	Medications	Angiotensin receptor blocker medication use (from PIN database) within 180 days prior to date of index visit
ACEI	Medications	Angiotensin-converting enzyme medication use (from PIN database) within 180 days prior to date of index visit
Entresto	Medications	Entresto medication use (from PIN database) within 180 days prior to date of index visit
Beta-blocker	Medications	Beta-blocker medication use (from PIN database) within 180 days prior to date of index visit
Ivabradine	Medications	Ivabradine medication use (from PIN database) within 180 days prior to date of index visit
Calcium channel blocker	Medications	Calcium channel blocker (dihydropyridine or non-dihydropyridine) medication use (from PIN database) within 180 days prior to date of index visit
Dihydropyridine calcium channel blocker	Medications	Dihydropyridine calcium channel blocker medication use (from PIN database) within 180 days prior to date of index visit
Non-dihydropyridine calcium channel blocker	Medications	Non-dihydropyridine calcium channel blocker medication use (from PIN database) within 180 days prior to date of index visit
Loop diuretic	Medications	Loop diuretic medication use (from PIN database) within 180 days prior to date of index visit

Thiazide diuretic	Medications	Thiazide diuretic medication use (from PIN database) within 180 days prior to date of index visit
Non-thiazide diuretic	Medications	Low-ceiling non-thiazide diuretic medication use (from PIN database) within 180 days prior to date of index visit
Metolazone	Medications	Metolazone medication use (from PIN database) within 180 days prior to date of index visit
Indapamide	Medications	Indapamide medication use (from PIN database) within 180 days prior to date of index visit
MRA	Medications	Potassium-sparing diuretic medication use (from PIN database) within 180 days prior to date of index visit
Vasopressin	Medications	Vasopressin antagonist diuretic medication use (from PIN database) within 180 days prior to date of index visit
Anti-arrhythmic medication	Medications	Anti-arrhythmic medication use (from PIN database) within 180 days prior to date of index visit
Amiodarone	Medications	Amiodarone medication use (from PIN database) within 180 days prior to date of index visit
Disopyramide	Medications	Disopyramide medication use (from PIN database) within 180 days prior to date of index visit
Digoxin	Medications	Digoxin medication use (from PIN database) within 180 days prior to date of index visit
Mavacamten	Medications	Mavacamten medication use (from PIN database) within 180 days prior to date of index visit
Amyloid therapeutics	Medications	Amyloid therapeutic medication use (from PIN database) within 180 days prior to date of index visit
Tafamidis	Medications	Tafamidis medication use (from PIN database) within 180 days prior to date of index visit
Diflunisal	Medications	Diflunisal medication use (from PIN database) within 180 days prior to date of index visit
Patisiran	Medications	Patisiran medication use (from PIN database) within 180 days prior to date of index visit
Inotersen	Medications	Inotersen medication use (from PIN database) within 180 days prior to date of index visit
Vutrisiran	Medications	Vutrisiran medication use (from PIN database) within 180 days prior to date of index visit
Lipid any	Medications	Lipid-lowering (any) medication use (from PIN database) within 180 days prior to date of index visit

Statin	Medications	Statin medication use (from PIN database) within 180 days prior to date of index visit
Fibrate	Medications	Fibrate medication use (from PIN database) within 180 days prior to date of index visit
Ezetimibe	Medications	Ezetimibe medication use (from PIN database) within 180 days prior to date of index visit
PCKSK9	Medications	PCSK9 inhibitor medication use (from PIN database) within 180 days prior to date of index visit
Lipid - other	Medications	Other lipid-lowering (other than statins, fibrates, ezetimibe, and PCSK9 inhibitors) medication use (from PIN database) within 180 days prior to date of index visit
Any glucose	Medications	Glucose-lowering (any) medication use (from PIN database) within 180 days prior to date of index visit
Insulin	Medications	Insulin medication use (from PIN database) within 180 days prior to date of index visit
GLP-1 agonist	Medications	GLP-1 agonist medication use (from PIN database) within 180 days prior to date of index visit
Hypoglycemic medication	Medications	Oral hypoglycemic (any) medication use (from PIN database) within 180 days prior to date of index visit
Metformin	Medications	Biguanide medication use (from PIN database) within 180 days prior to date of index visit
Alpha glucosidase inhibitor	Medications	Alpha-glucosidase inhibitor medication use (from PIN database) within 180 days prior to date of index visit
DPP-4 inhibitor	Medications	DPP-4 inhibitor medication use (from PIN database) within 180 days prior to date of index visit
SGLT-2 inhibitor	Medications	SGLT2 inhibitor medication use (from PIN database) within 180 days prior to date of index visit
Thiazolidine	Medications	Thiazolidine medication use (from PIN database) within 180 days prior to date of index visit
Repaglinide	Medications	Repaglinide medication use (from PIN database) within 180 days prior to date of index visit
Sulphonyl urea	Medications	Sulfonylurea medication use (from PIN database) within 180 days prior to date of index visit
Other hypoglycemic medication	Medications	Other oral hypoglycemic (other than biguanide, alpha-glucosidase inhibitor, DPP-4 inhibitor, SGLT2 inhibitor, thiazolidine, repaglinide, and sulfonylurea) medication use (from PIN

		database) within 180 days prior to date of index visit
Bupropion or Varenicline	Medications	Other oral hypoglycemic (other than biguanide, alpha-glucosidase inhibitor, DPP-4 inhibitor, SGLT2 inhibitor, thiazolidine, repaglinide, and sulfonylurea) medication use (from PIN database) within 180 days prior to date of index visit
Nicotine replacement therapy	Medications	Oral smoking cessation medication use (from PIN database) within 180 days prior to date of index visit
Hypertension combined	Medical History	Hypertension detected by either: 1) ICD-10 coding in either DAD or NACRS databases prior to index visit. 2) Thiazide diuretic or low-ceiling non-thiazide diuretic use recorded in PIN database within 180 days prior to index visit
Diabetes combined	Medical History	Diabetes mellitus detected by either: 1) ICD-10 coding in either DAD or NACRS databases prior to index visit. 2) Glucose-lowering medication use recorded in PIN database within 180 days prior to index visit. 3) HbA1C >= 6.5 within 3 years prior to index visit
Dyslipidemia combined	Medical History	Dyslipidemia detected by either: 1) ICD-10 coding in either DAD or NACRS databases prior to index visit. 2) Lipid-lowering medication use recorded in PIN database within 180 days prior to index visit. 3) LDL-cholesterol >= 3.5 within 3 years prior to index visit. 3) Triglycerides >= 1.7 within 3 years prior to index visit
Prior CAD	Medical History	ICD-10 coding of coronary artery disease in either DAD or NACRS databases at any time prior to index visit
Dilated cardiomyopathy	Medical History	ICD-10 coding of dilated cardiomyopathy in either DAD or NACRS databases at any time prior to index visit
Hypertrophic cardiomyopathy	Medical History	ICD-10 coding of hypertrophic cardiomyopathy in either DAD or NACRS databases at any time prior to index visit
AV block 3rd degree	Medical History	ICD-10 coding of 3rd degree (complete) heart block in either DAD or NACRS databases at any time prior to index visit
Acute myocarditis	Medical History	ICD-10 coding of acute myocarditis in either DAD or NACRS databases at any time prior to index visit

Acute pericarditis	Medical History	ICD-10 coding of acute pericarditis in either DAD or NACRS databases at any time prior to index visit
Aortic aneurysm	Medical History	ICD-10 coding of aortic aneurysm in either DAD or NACRS databases at any time prior to index visit
Aortic dissection	Medical History	ICD-10 coding of aortic dissection in either DAD or NACRS databases at any time prior to index visit
Pulmonary hypertension	Medical History	ICD-10 coding of pulmonary hypertension in either DAD or NACRS databases at any time prior to index visit
Primary PHTN	Medical History	ICD-10 coding of primary pulmonary hypertension in either DAD or NACRS databases at any time prior to index visit
Secondary PHTN	Medical History	ICD-10 coding of secondary pulmonary hypertension in either DAD or NACRS databases at any time prior to index visit
Amyloid	Medical History	ICD-10 coding of amyloidosis in either DAD or NACRS databases at any time prior to index visit
Carpal tunnel	Medical History	ICD-10 coding of carpal tunnel syndrome in either DAD or NACRS databases at any time prior to index visit
Chronic obstructive pulmonary disease	Medical History	ICD-10 coding of COPD in either DAD or NACRS databases at any time prior to index visit
Obstructive sleep apnea	Medical History	ICD-10 coding of obstructive sleep apnea in either DAD or NACRS databases at any time prior to index visit
Hyperthyroid	Medical History	ICD-10 coding of hyperthyroidism in either DAD or NACRS databases at any time prior to index visit
Hypothyroid	Medical History	ICD-10 coding of hypothyroidism in either DAD or NACRS databases at any time prior to index visit
Muscular dystrophy	Medical History	ICD-10 coding of muscular dystrophy in either DAD or NACRS databases at any time prior to index visit
Rheumatoid arthritis	Medical History	ICD-10 coding of rheumatoid arthritis in either DAD or NACRS databases at any time prior to index visit
Lupus	Medical History	ICD-10 coding of systemic lupus erythematosus in either DAD or NACRS databases at any time prior to index visit

JCM_alec_chunta

Scleroderma	Medical History	ICD-10 coding of scleroderma in either DAD or NACRS databases at any time prior to index visit
Sarcoidosis	Medical History	ICD-10 coding of sarcoidosis in either DAD or NACRS databases at any time prior to index visit
Cancer	Medical History	ICD-10 coding of cancer in either DAD or NACRS databases at any time prior to index visit
Mitral valves surgical intervention	Medical History	Mitral valve procedure (repair or replacement) by CCI coding prior to index visit
Mitral valve replacement	Medical History	Mitral valve replacement by CCI coding prior to index visit
Mitral valve repair	Medical History	Mitral valve repair by CCI coding prior to index visit
Aortic surgical intervention	Medical History	Aortic valve procedure (repair or replacement) by CCI coding prior to index visit
Aortic valve replacement	Medical History	Aortic valve replacement by CCI coding prior to index visit
Aortic valve repair	Medical History	Aortic valve repair by CCI coding prior to index visit
Tricuspid valve surgical intervention	Medical History	Tricuspid valve procedure (repair or replacement) by CCI coding prior to index visit
Tricuspid valve replacement	Medical History	Tricuspid valve replacement by CCI coding prior to index visit
Tricuspid valve repair	Medical History	Tricuspid valve repair by CCI coding prior to index visit
Pulmonary valve surgical intervention	Medical History	Pulmonary valve procedure (repair or replacement) by CCI coding prior to index visit
Pulmonary valve replacement	Medical History	Pulmonary valve replacement by CCI coding prior to index visit
Pulmonary valve repair	Medical History	Pulmonary valve repair by CCI coding prior to index visit
Heart transplant	Medical History	Heart transplantation by CCI coding prior to index visit
LVAD implementation	Medical History	Left ventricular assist device (LVAD) implantation by CCI coding prior to index visit

Permanent pacemaker device implantation	Medical History	Permanent pacemaker implantation by CCI coding prior to index visit
Single permanent pacemaker device implantation	Medical History	Single-chamber permanent pacemaker implantation by CCI coding prior to index visit
Dual permanent pacemaker device implantation	Medical History	Dual chamber permanent pacemaker implantation by CCI coding prior to index visit
CRT-Pacing	Medical History	CRT-P implantation by CCI coding prior to index visit
Implantable cardioverter defibrillator	Medical History	ICD implantation by CCI coding prior to index visit
CRT-defibrillator	Medical History	CRT-D implantation by CCI coding prior to index visit
Prior CABG surgery	Medical History	Prior CABG from APPROACH database
Heart Failure Hospitalization	Medical History	Heart failure hospitalization by ICD-10 coding prior to index visit
Any acute coronary syndrome	Medical History	Any acute coronary syndrome by ICD-10 coding prior to index visit
Acute myocardial infarction	Medical History	Acute myocardial infarction by ICD-10 coding prior to index visit
Unstable angina	Medical History	Unstable angina by ICD-10 coding prior to index visit
Other acute coronary syndrome	Medical History	Other acute coronary syndrome (other than acute myocardial infarction and unstable angina) by ICD-10 coding prior to index visit
Atrial fibrillation or atrial flutter	Medical History	Any documented atrial fibrillation or flutter by ICD-10 coding prior to index visit
Atrial fibrillation	Medical History	Any documented atrial fibrillation by ICD-10 coding prior to index visit
Atrial flutter	Medical History	Any documented atrial flutter by ICD-10 coding prior to index visit
Ventricular tachycardia	Medical History	Any ventricular tachycardia by ICD-10 coding prior to index visit
Survived sudden cardiac arrest	Medical History	Survived sudden cardiac arrest by ICD-10/CCI coding prior to index visit

Stroke	Medical History	Any acute stroke by ICD-10 coding prior to index visit
Acute ischemic stroke	Medical History	Acute ischemic stroke by ICD-10 coding prior to index visit
Acute hemorrhagic stroke	Medical History	Acute hemorrhagic stroke by ICD-10 coding prior to index visit
Transient ischemic attack	Medical History	TIA by ICD-10 coding prior to index visit
Chronic kidney disease	Medical History	
Prior PCI	Medical History	Prior PCI from APPROACH database

Table S2: Summary of dataset sources used to obtain data for internal and external cohorts

Source	Information	Variable Example
Discharge Abstract Database	Demographics, administrative data, clinical information, and diagnoses.	Age, Prior CAD
National Ambulatory Care Reporting System	ICD-10 codes for clinical data.	Hypertension, Diabetes, Dyslipidemia.
Pharmaceutical Information Network	Medications data	Anti-platelet, Statin, Beta blocker.
APPROACH	Clinical data for patients undergoing cardiac procedures	Prior PCI, Prior CABG.
AHS Labs	Laboratory data	Hemoglobin, Sodium, Potassium.

JCM_alec_chunta

Table S3: Values with missingness in the internal and external cohorts;

Variable	Category	Internal Missing (n)	Internal Missing (%)	External Missing (n)	External Missing (%)
Age	Demographics	0	0	0	0
Male	Demographics	0	0	0	0
Alanine transaminase	Labs	745	8.4	2814	26.6
Albumin	Labs	3985	45.2	6340	59.8
Alkaline Phosphatase	Labs	2937	33.3	4246	40.1
Aspartate transaminase	Labs	6826	77.4	7944	75
Blood iron	Labs	2180	24.7	4951	46.7
Blood urea	Labs	3788	42.9	5473	51.7
Chloride	Labs	435	4.9	2674	25.2
Closest Hemoglobin A1C	Labs	808	9.2	4688	44.2
Closest High-density lipoprotein	Labs	608	6.9	3464	32.7
Closest fasting blood glucose	Labs	5106	57.9	4394	41.5
Closest lipid cholesterol	Labs	610	6.9	3427	32.3
Closest low-density lipoprotein	Labs	673	7.6	3544	33.4
Closest random blood glucose	Labs	2632	29.8	5731	54.1
Creatinine	Labs	152	1.7	2318	21.9
Creatine kinase	Labs	4832	54.8	5857	55.3
Hematocrit	Labs	236	2.7	2184	20.6
Hemoglobin	Labs	239	2.7	2174	20.5
Highest Hemoglobin A1C	Labs	808	9.2	4677	44.1
Highest fasting blood glucose	Labs	5106	57.9	4391	41.4
Highest lipid cholesterol	Labs	610	6.9	3400	32.1
Highest low-density lipoprotein	Labs	673	7.6	3518	33.2

Highest random blood glucose	Labs	2632	29.8	5691	53.7
Lowest High-density lipoprotein	Labs	608	6.9	3457	32.6
Partial thromboplastin time	Labs	7278	82.5	6973	65.8
Platelet count	Labs	241	2.7	2177	20.5
Potassium	Labs	428	4.9	2627	24.8
Red cell distribution width	Labs	482	5.5	3326	31.4
Serum Ferritin	Labs	5000	56.7	8702	82.1
Sodium	Labs	783	8.9	4609	43.5
Thyroid stimulating hormone	Labs	1148	13	3418	32.3
Total bilirubin	Labs	4240	48.1	5285	49.9
Total iron binding capacity	Labs	4986	56.5	8701	82.1
Triglycerides closest	Labs	611	6.9	6328	59.7
Triglycerides highest	Labs	611	6.9	6324	59.7
Urinary albumin/Cr ratio	Labs	6202	70.3	7793	73.5
White blood cell count	Labs	243	2.8	2182	20.6
Chronic Kidney Disease	Medical History	2010	22.8	2318	21.9

Table S4: Missingness in the Calgary (internal) and Ottawa (external) cohorts for secondary testing

Variable	Category	Internal Missing (n)	Internal Missing (%)	External Missing (n)	External Missing (%)
Age	Demographics	0	0	0	0
Male	Demographics	0	0	0	0
Any Self reported chest pain	Indication	5667	64.2	162	1.6
Any self report shortness of breath	Indication	5667	64.2	448	4.5
Albumin	Labs	3985	45.2	8753	88.8
Chloride	Labs	783	8.9	6921	70.2

Closest Hemoglobin A1C	Labs	808	9.2	8065	81.8
Closest High density lipoprotein	Labs	608	6.9	8862	89.9
Closest lipid cholesterol	Labs	610	6.9	8850	89.8
Closest low density lipoprotein	Labs	673	7.6	8878	90
Creatinine	Labs	152	1.7	651	6.6
Hematocrit	Labs	236	2.7	6700	68
Hemoglobin	Labs	239	2.7	6700	68
Platelet count	Labs	241	2.7	6701	68
Potassium	Labs	428	4.9	6820	69.2
Sodium	Labs	435	4.9	6818	69.2
Triglycerides closest	Labs	611	6.9	8871	90
White blood cell count	Labs	243	2.8	6700	68
Acute Myocardial Infarction	Medical History	0	0	150	1.5
Diabetes	Medical History	0	0	142	1.4
Dyslipidemia	Medical History	0	0	162	1.6
Heart Failure Hospitalization	Medical History	0	0	129	1.3
Hypertension	Medical History	0	0	104	1.1
Peripheral Arterial Disease	Medical History	0	0	160	1.6
Prior CABG Surgery	Medical History	4	0	160	1.6
Prior PCI	Medical History	4	0	163	1.7
Transient ischemic attack	Medical History	0	0	160	1.6
ARB	Medication	0	0	3054	31
All diuretics	Medication	0	0	150	1.5
Anti-arrhythmic medication	Medication	0	0	3208	32.5
Anti-platelet ASA agent	Medication	0	0	144	1.5
Any Nitrates	Medication	0	0	163	1.7
Beta-blocker	Medication	0	0	133	1.3
Calcium channel Blocker	Medication	0	0	155	1.6
Oral anticoagulant use	Medication	0	0	3187	32.3
Statin	Medication	0	0	1889	19.2

Table S5: Population characteristics, Calgary and Ottawa;

Variable	Calgary (n=8,823)	Ottawa (n=9,859)	p-value
Male, n (%)	5084 (57.6%)	5491 (55.7%)	0.008
Age, median (IQR)	59 (49-66)	59 (51-67)	<0.001
Subsequent ICA, n (%)	1161 (13.2%)	294 (3.0%)	<0.001

**Indication**, n (%)			
Chest pain	1563 (17.7%)	5085 (51.6%)	0.005
**Medical History**, n (%)			
Diabetes	2031 (23.0%)	1459 (14.8%)	<0.001
Hypertension	3183 (36.1%)	4819 (48.9%)	<0.001
Dyslipidemia	6667 (75.6%)	4732 (48.0%)	<0.001
Prior PCI	253 (2.9%)	382 (3.9%)	<0.001
**Medications**, n (%)			
Anti-Platelet	645 (7.3%)	1883 (19.4%)	<0.001
Beta Blocker	5628 (63.8%)	3805 (39.1%)	<0.001
Anti-Coagulant	695 (7.9%)	1282 (19.2%)	<0.001
**Labs**, median (IQR)			
Closest HDL	1.26 (1.03–1.54)	1.21 (1-1.49)	<0.001
Triglycerides Closest	1.39 (0.98-2.02)	1.31 (0.96-1.9625)	0.197
White Blood Cells	6.5 (5.4-7.8)	7 (5.8-8.6)	<0.001

Table S6: Classification consequences of the reduced XGBoost model per 1000 patients at selected threshold probabilities.

**Cohort**	**Threshold probability**	**True positives**	**False positives**	**False negatives**	**True negatives**	**PPV**	**NPV**	**Sensitivity**	**Specificity**
Calgary	5.0%	131	850	0	18	0.134	0.988	0.998	0.021
Calgary	10.0%	108	499	23	369	0.178	0.941	0.823	0.425
Calgary	13.4%	86	307	46	561	0.218	0.925	0.652	0.647
Calgary	15.0%	74	244	57	624	0.233	0.916	0.565	0.718
Calgary	20.0%	44	106	88	763	0.292	0.897	0.332	0.878
Calgary	30.0%	11	20	120	849	0.359	0.876	0.084	0.977
Edmonton	5.0%	119	871	0	9	0.120	0.951	0.996	0.011
Edmonton	10.0%	96	476	24	405	0.168	0.944	0.799	0.460
Edmonton	13.4%	72	298	48	582	0.195	0.925	0.603	0.662
Edmonton	15.0%	59	216	61	664	0.215	0.916	0.494	0.755
Edmonton	20.0%	29	78	91	802	0.268	0.898	0.239	0.911
Edmonton	30.0%	6	14	114	866	0.303	0.884	0.050	0.984

Counts are rounded from the classification rates and may not sum to exactly 1000. A threshold probability of 0.134 is the Youden-derived threshold applied in Tables 2 and 5;

sensitivity, specificity, PPV and NPV at this threshold correspond to the values reported there. PPV: positive predictive value; NPV: negative predictive value.

Figure S1: Correlation heatmap for the internal cohort (Calgary)

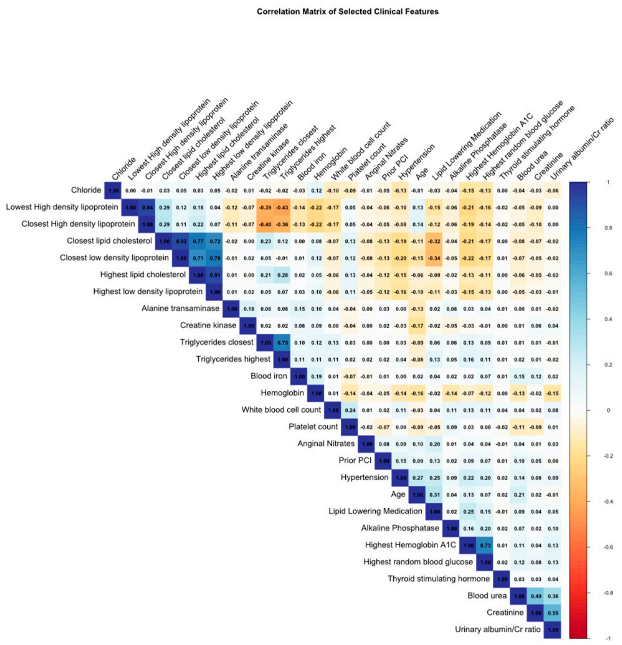

Pearson correlation heatmap across the gain-ranked candidate features. Variable pairs exceeding r > 0.85 were identified and the lower gain-ranked feature of each pair was removed to reduce multicollinearity before building the parsimonious model.

Figure S2: Correlation heatmap for the internal cohort (Calgary) in secondary testing

JCM_alec_chunta

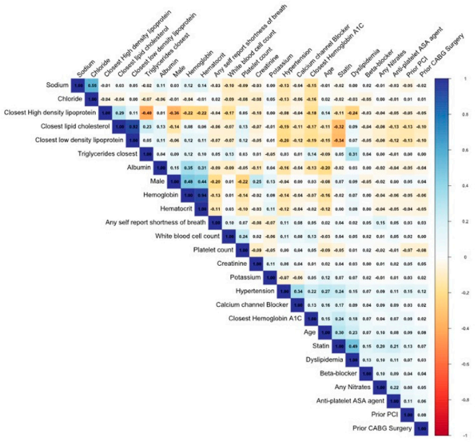

Pearson correlation heatmap across the gain-ranked candidate features in the 49-variable secondary analysis. Pairs with r > 0.85 were filtered, retaining the higher gain-ranked feature of each pair.

JCM_alec_chunta

Figure S3: Gain-based feature importance for internal training (Calgary) in secondary testing

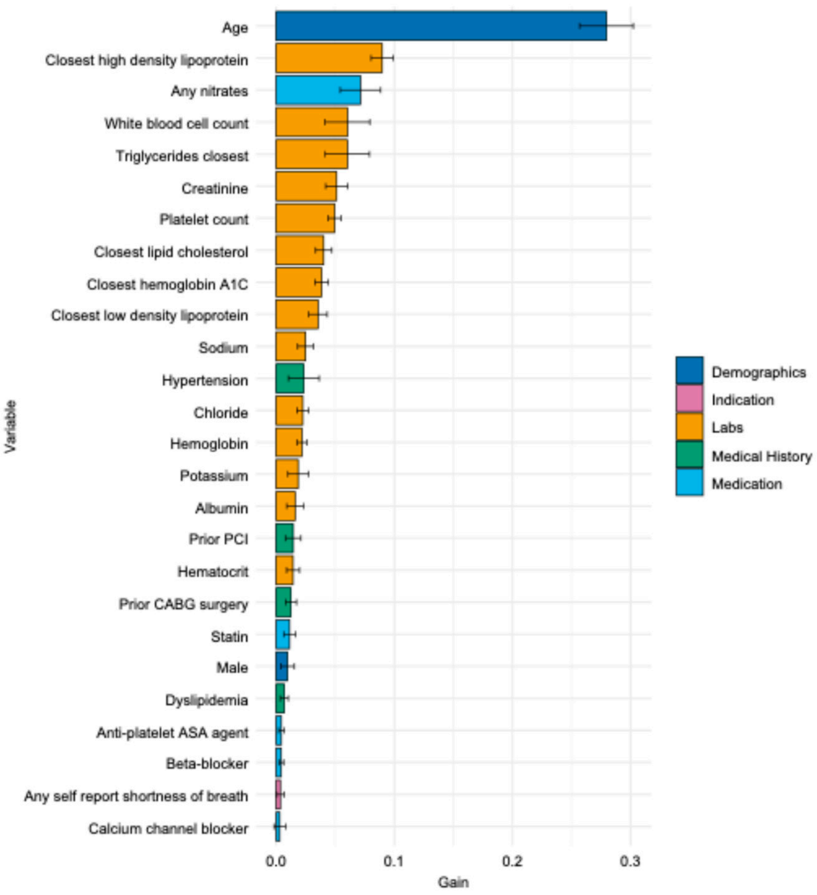

Mean gain-based importance for each variable in the internal model of the secondary analysis, ranked in descending order across cross-validation folds. The highest-ranked predictors of subsequent ICA were age, closest high density lipoprotein, and nitrate medication usage.

Figure S4: Prediction performance of the full and reduced XGBoost models on the internal cohort (Calgary) in secondary testing

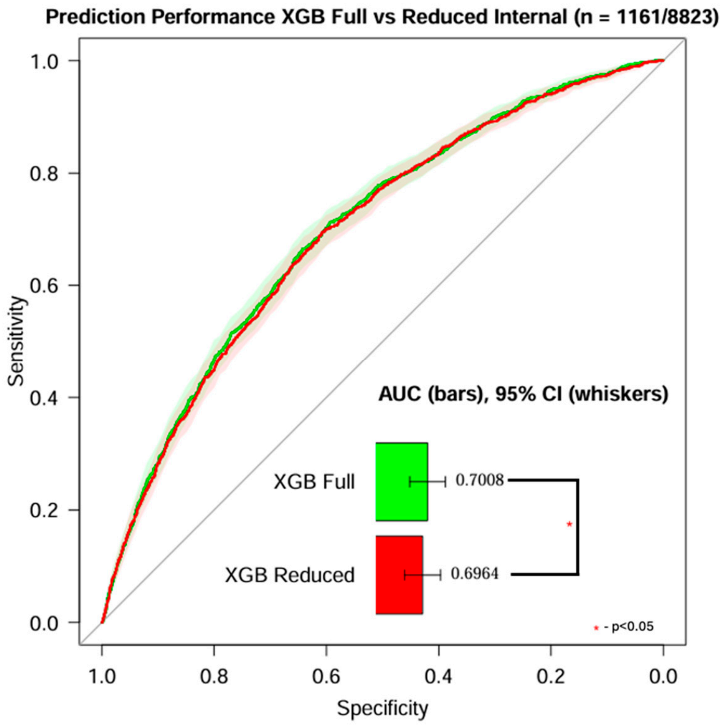

AUC/ROC curves for the full and reduced XGBoost models. Internal discrimination was statistically different (full AUC 0.701 versus reduced AUC 0.696; DeLong p = 0.0013), indicating that feature reduction had an effect on discrimination in the development cohort. The diagonal line denotes chance performance.

Figure S5: Model calibration for the full and reduced XGBoost models on the internal cohort (Calgary) in secondary testing

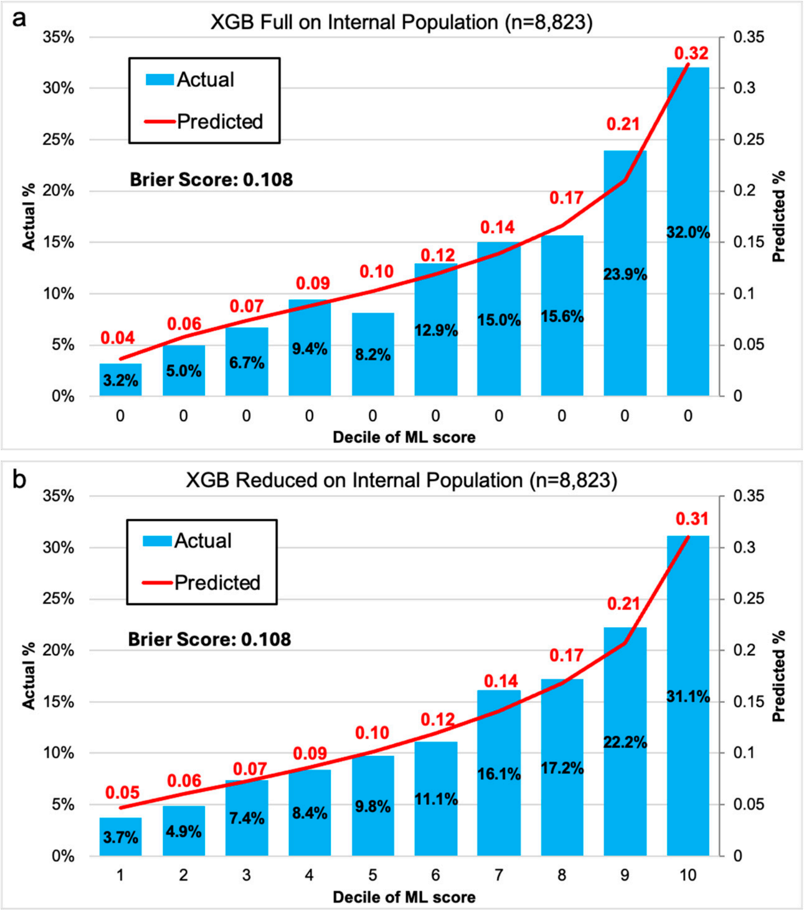

Calibration plots (a: full; b: reduced) of predicted versus observed proportion of ICA referrals by decile of predicted risk. Brier scores: full (0.108) versus reduced (0.108).

JCM_alec_chunta

Figure S6: Sequential AUC progression in the internal cohort (Calgary) in secondary testing

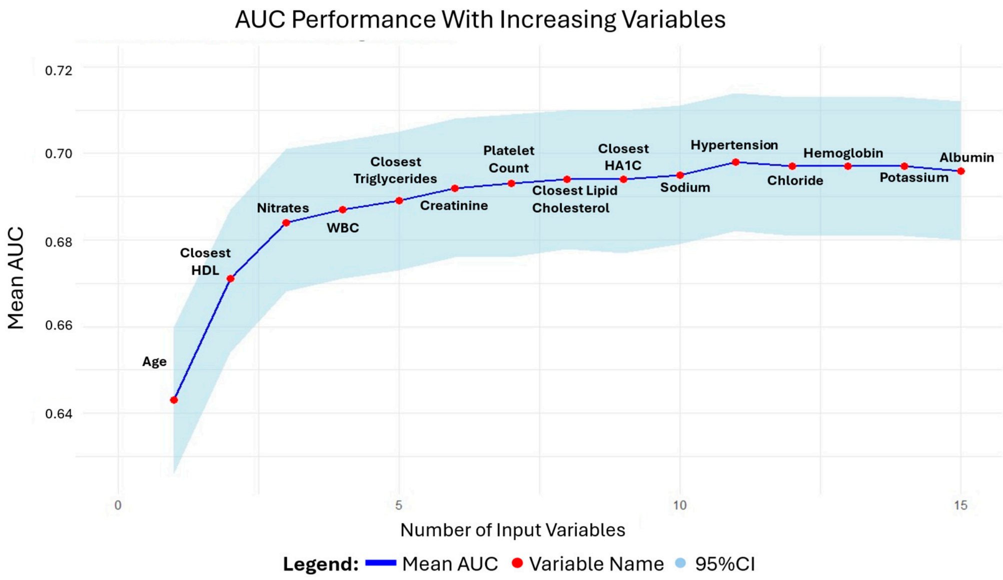

Sequential AUC as each top-ranked variable is added in descending order of gain importance. Performance plateaued at a final AUC of 0.696 after 15 variables but achieved a maximum AUC of 0.698 after the addition of 11 variables.

Figure S7: Decision curve analysis across the full range of threshold probabilities (0 to 95%) for the full and reduced XGBoost models.

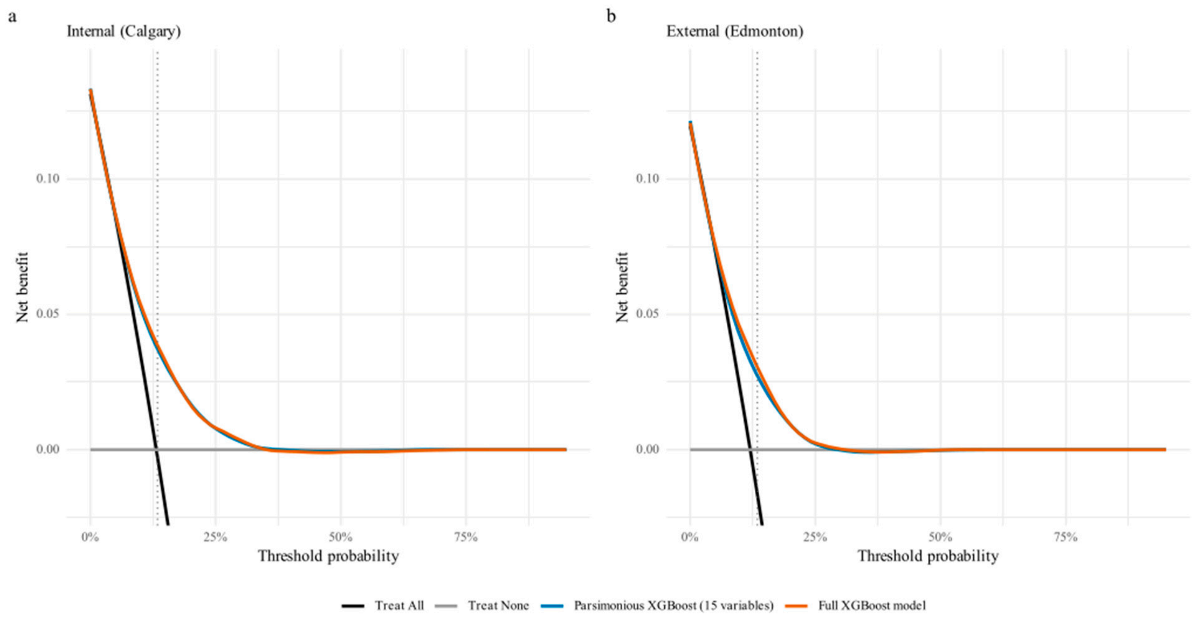

(a) Internal cohort (Calgary); (b) external cohort (Edmonton). Above approximately 45% all curves converge, as predicted probabilities exceeded 0.50 in only 8 of 8,823 internal and 3 of 10,596 external patients.

JCM_alec_chunta

Figure S8: SHAP Beeswarm plot showing feature rank and directional SHAP values for the top features in the external cohort (Edmonton)

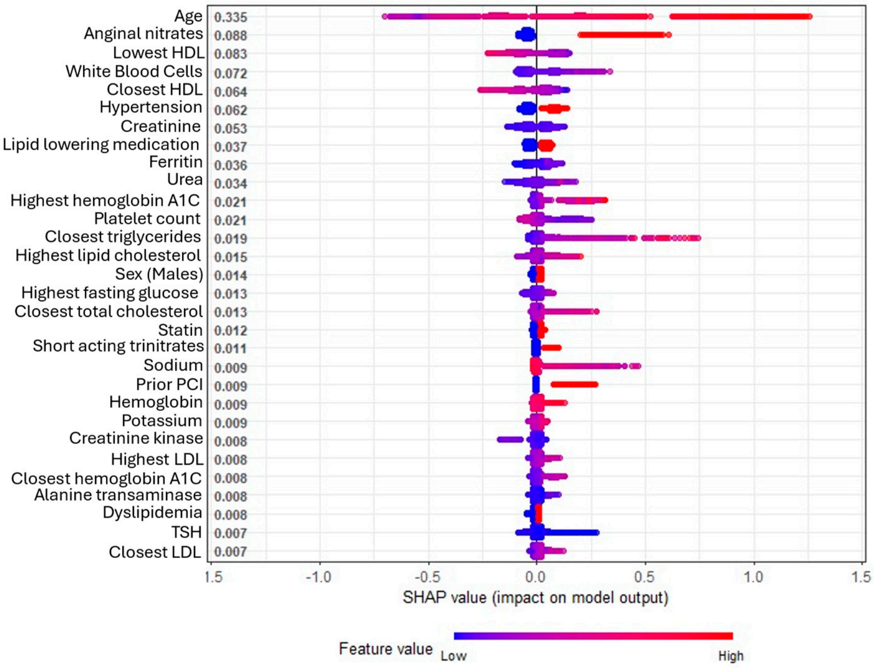

SHAP beeswarm plot: each point is one patient, horizontal position is the SHAP value (contribution to predicted ICA-referral probability), and colour encodes feature value (low to high). Features are ranked by mean absolute SHAP value. Higher age, Anginal nitrate usage and lower HDL contribute most to the prediction of patients receiving a subsequent ICA.

JCM_alec_chunta

Figure S9: SHAP beeswarm plot for the external cohort (Ottawa) in secondary testing

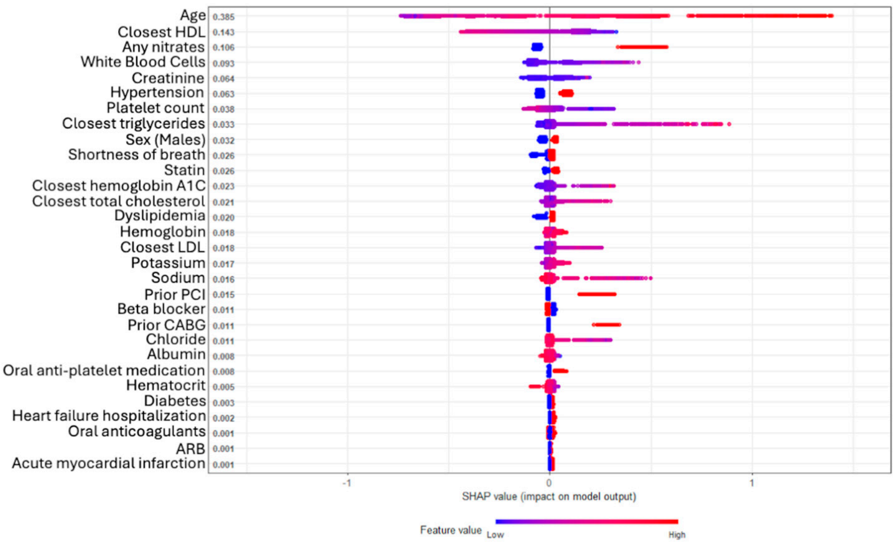

Features are ranked by mean absolute SHAP value, showing feature rank and the direction of each feature's effect on predicted ICA-referral probability in Ottawa. Higher age, lower HDL cholesterol, and nitrate usage contributed the most to the prediction of which patients should receive a subsequent ICA.

JCM_alec_chunta

Figure S10: Prediction performance of the full and reduced XGBoost models on the external cohort (Ottawa) in secondary testing

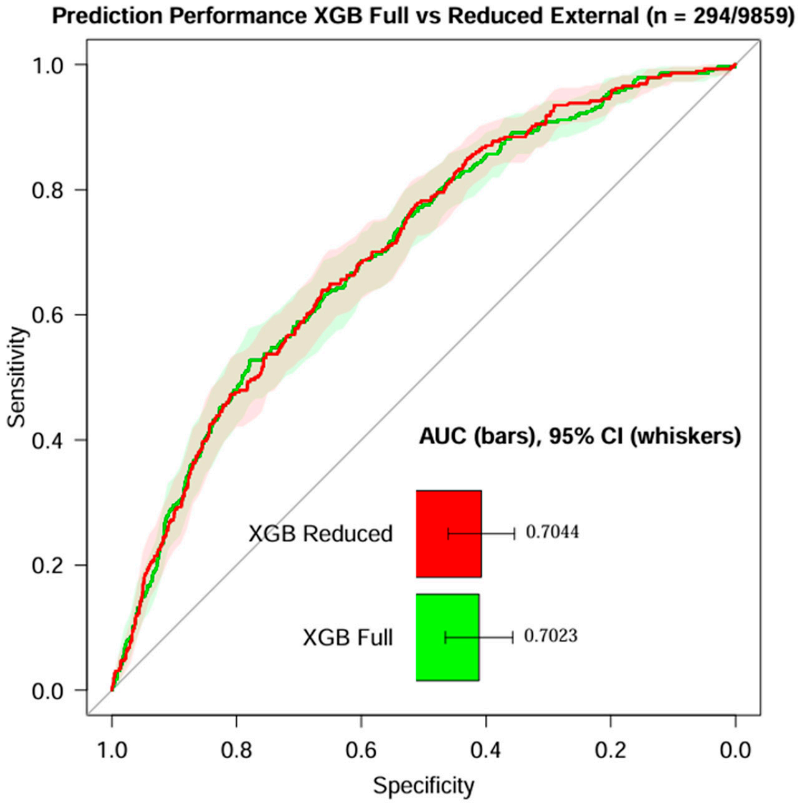

AUC/ROC curves for the full and reduced XGBoost models in the Ottawa cohort. Full AUC (0.704) versus reduced AUC (0.702); p = 0.664.

Figure S11: Model calibration for the full and reduced XGBoost models on the external cohort (Ottawa) in secondary testing

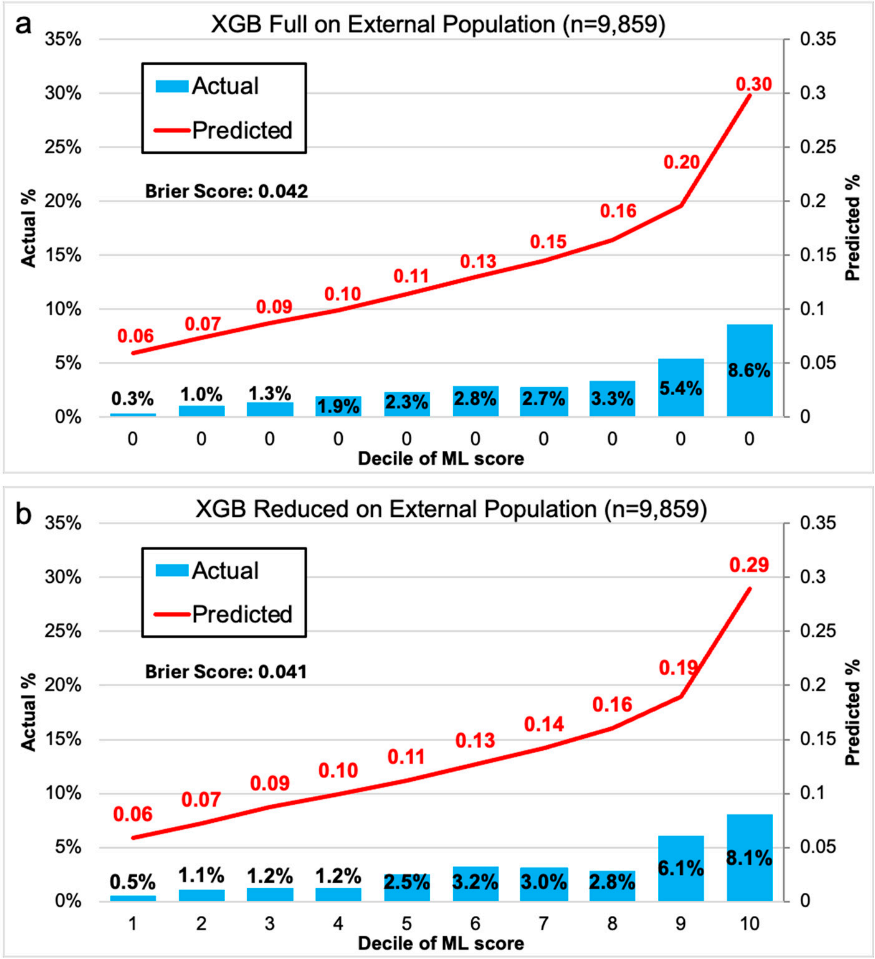

The reduced model had a lower but non-significant Brier score compared to the full model. The low internal-to-external prevalence shift (13.2% → 3.0%) manifests as calibration-in-the-large drift while discrimination is preserved. Brier scores: XGB full (0.042) reduced (0.041).

Figure S12: Sequential AUC progression in the external (Ottawa) cohort for secondary testing.

JCM_alec_chunta

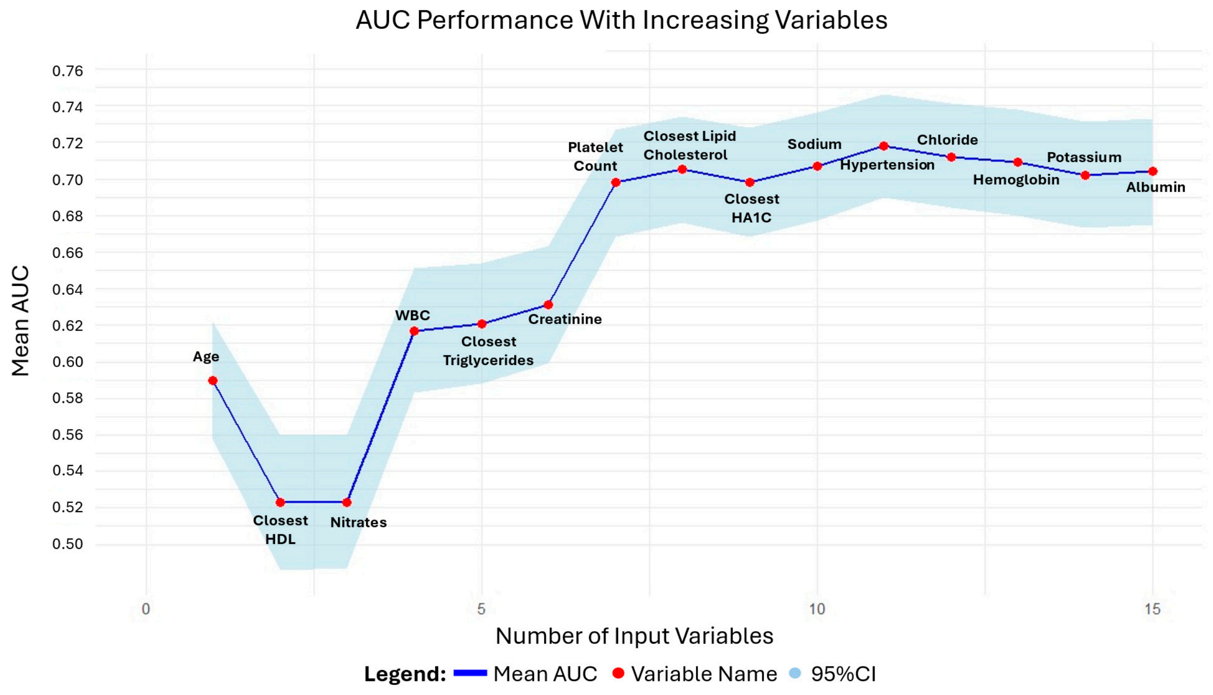

Sequential AUC as each top-ranked variable is added in descending order of gain importance. The reduced model achieved a highest AUC of 0.718 after 11 variables and reached a final AUC of 0.704 after 15 variables.
